# A high-content EMT screen identifies multiple receptor tyrosine kinase inhibitors with activity on TGFβ receptor

**DOI:** 10.18632/oncotarget.8418

**Published:** 2016-03-27

**Authors:** Carina Lotz-Jenne, Urs Lüthi, Sabine Ackerknecht, François Lehembre, Tobias Fink, Manuel Stritt, Matthias Wirth, Simona Pavan, Ruben Bill, Urs Regenass, Gerhard Christofori, Nathalie Meyer-Schaller

**Affiliations:** ^1^ Actelion Pharmaceuticals Ltd., Allschwil, Switzerland; ^2^ Department of Biomedicine, University of Basel, Basel, Switzerland; ^3^ Current address: European Patent Office, Rijswijk, The Netherlands

**Keywords:** EMT, TGFβ, cytoskeleton, ROCK, receptor tyrosine kinase

## Abstract

An epithelial to mesenchymal transition (EMT) enables epithelial tumor cells to break out of the primary tumor mass and to metastasize. Understanding the molecular mechanisms driving EMT in more detail will provide important tools to interfere with the metastatic process. To identify pharmacological modulators and druggable targets of EMT, we have established a novel multi-parameter, high-content, microscopy-based assay and screened chemical compounds with activities against known targets. Out of 3423 compounds, we have identified 19 drugs that block transforming growth factor beta (TGFβ)-induced EMT in normal murine mammary gland epithelial cells (NMuMG). The active compounds include inhibitors against TGFβ receptors (TGFBR), Rho-associated protein kinases (ROCK), myosin II, SRC kinase and uridine analogues. Among the EMT-repressing compounds, we identified a group of inhibitors targeting multiple receptor tyrosine kinases, and biochemical profiling of these multi-kinase inhibitors reveals TGFBR as a thus far unknown target of their inhibitory spectrum. These findings demonstrate the feasibility of a multi-parameter, high-content microscopy screen to identify modulators and druggable targets of EMT. Moreover, the newly discovered “off-target” effects of several receptor tyrosine kinase inhibitors have important consequences for *in vitro* and *in vivo* studies and might beneficially contribute to the therapeutic effects observed *in vivo*.

## INTRODUCTION

An epithelial to mesenchymal transition (EMT) is a critical event during embryonic development and wound healing, when cell motility is required [1]. In recent years, the important role of EMT has been extended to cancer cell migration and invasion, both processes being defined as central hallmarks of cancer [2]. EMT can be induced by a variety of growth factors, most importantly by transforming growth factor beta (TGFβ). Upon transition to a mesenchymal state, dedifferentiated cancer cells break out from the primary tumor mass, invade the surrounding tissue and seed distant metastases through the blood or lymphatic system. Cancer cells that have undergone an EMT keep their plasticity and can either revert back to an epithelial state by a process termed mesenchymal to epithelial transition (MET) or stay in a dormant state [3]. MET has been shown to favor the outgrowth of metastases at distant sites. This plasticity is associated with cancer stem cell-like features and increased resistance to chemotherapy [4, 5]. Moreover, while the role of EMT to the actual metastatic process has recently been challenged by cell tracing experiments in transgenic mouse models of breast cancer, the contribution of EMT to the formation of metastasis under chemotherapy is substantial [6, 7]. A better understanding of how EMT is achieved will generate new strategies to prevent metastases formation and the outgrowth of therapy-resistant cancer stem cells in relapsing patients.

During TGFβ-induced EMT, epithelial cells undergo major morphological and functional changes to lose cell-cell contacts and apical-basal polarity while allowing for invasion of the surrounding tissue [8]. The loss of adherens and tight junction proteins, such as E-cadherin and zonula occludens 1 (ZO1), respectively, is accompanied by the increased expression of the mesenchymal proteins N-cadherin, fibronectin and vimentin. Interestingly, the loss of E-cadherin is both a cause and a consequence of EMT and is therefore a key driver for local tumor invasiveness and systemic cancer cell dissemination [9, 10]. Furthermore, the remodeling of a cortical actin cytoskeleton in epithelial cells to stress fibers and the formation of focal adhesions in mesenchymal cells together with the upregulation of integrins, cathepsins and metalloproteases enable cancer cells to gain migratory and invasive behaviors [11].

In the past, multiple strategies to interfere with EMT and its consequences have been envisaged including the specific killing of cancer stem cells that are more resistant to chemotherapy. Salinomycin, a potassium ionophore, and metformin, a standard diabetes drug, display selective toxicity towards breast cancer stem cells [12, 13]. Consequently, metformin has shown promising efficacy in combination with chemotherapy in tumor xenograft mouse models. Moreover, multiple screening approaches have been performed to identify new druggable targets in the EMT process or to find compounds that could revert mesenchymal cells back to an epithelial state with higher vulnerability to standard therapies. A high-throughput siRNA screen against a library of human kinases has been performed in human breast and lung cancer cell lines harboring a TGFBR1 promoter-reporter construct to find modulators of TGFβ signaling [14]. The reversion of mesenchymal breast cancer cells to a more epithelial state using EpCAM and E-cadherin as epithelial markers has been employed as readout in a siRNA screen for genes critical for MET [15]. Similarly, in colorectal or melanoma cell lines, multiple compound screens have been performed to find cells with increased E-cadherin expression [16, 17]. A vimentin promoter-reporter assay in mesenchymal MDA-MB-231 human breast cancer cells grown in spheroids has been used to screen for compounds that provoke an epithelial phenotype [18]. These screening approaches were dependent on a single marker for EMT progression and may therefore reduce the identification of compounds and targets involved in EMT phenotype modulation.

In the present study, we have aimed at the identification of modulators and druggable targets of EMT with potential therapeutic benefit for breast cancer patients. To this end, we have established a multi-parameter, high-content microscopy screen that captures in depth specific EMT-associated changes in response to TGFβ. Using cultured normal murine mammary epithelial cells (NMuMG), we have followed the transition from an epithelial to a mesenchymal state through the upregulation of the mesenchymal marker fibronectin and the formation of actin stress fibers and focal adhesions. In addition, we have scored TGFβ-induced cell cycle arrest by cell counting. Screening a library of compounds with reported biochemical activities, we have validated the screening system by the identification of TGFBR kinase inhibitors and identified multiple ROCK inhibitors whose potency in inhibiting EMT progression correlated with their *in vitro* biochemical as well as cellular activity against ROCK. In addition, we have found multiple receptor tyrosine kinase (RTK) inhibitors able to block EMT due to their thus far uncharacterized inhibition of TGFBR activity.

## RESULTS

### Setup of the high-content microscopy screen

**Figure 1:**
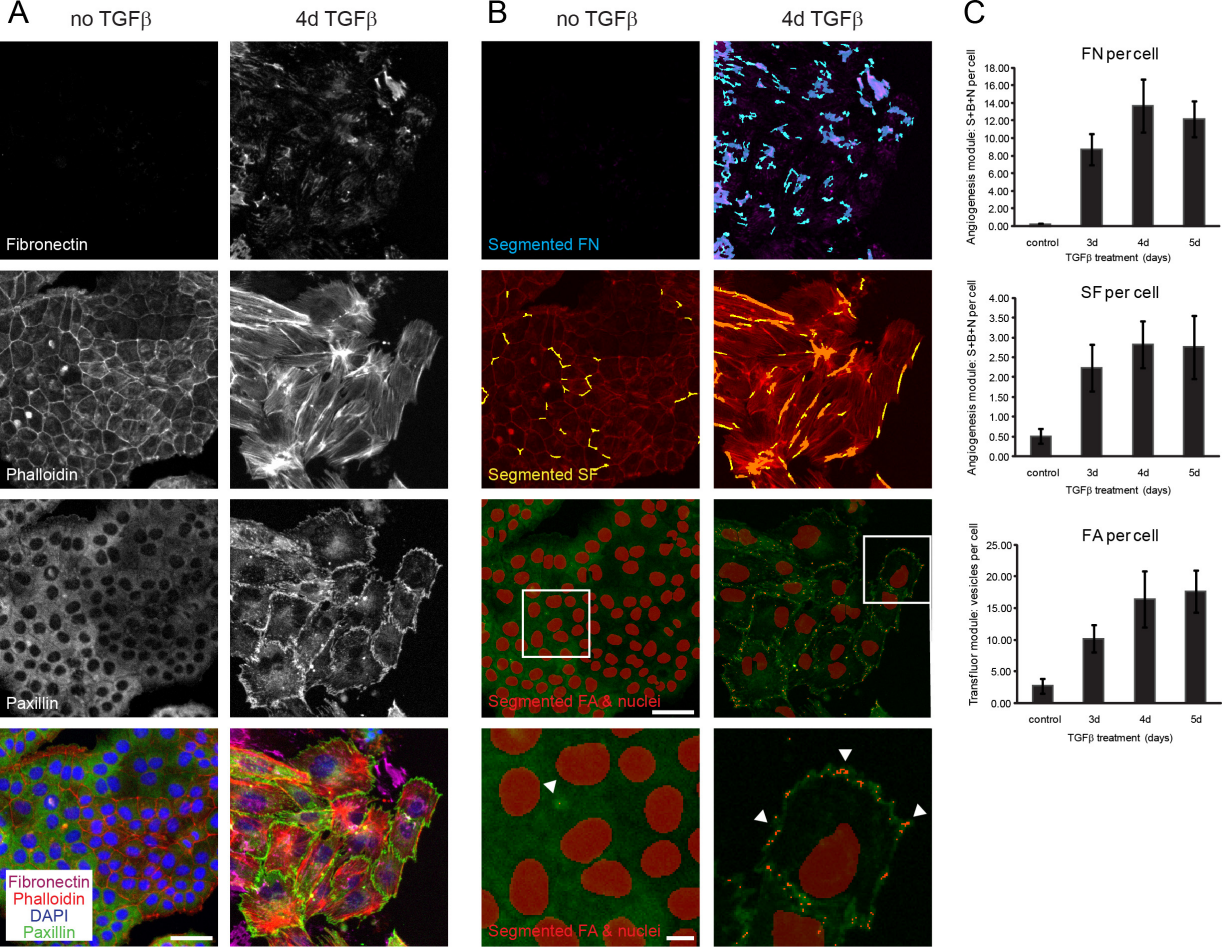
Segmentation and quantification of focal adhesions, actin stress fibers and fibronectin deposition as EMT readouts (**A**) NMuMG cells were treated with TGFβ for 3, 4 or 5 days or left untreated for 3 days, and cytoskeleton remodeling and fibronectin upregulation was followed by immunofluorescence stainings for fibronectin, for filamentous actin (phalloidin-568), for paxillin to stain focal adhesions and DAPI for nuclear staining (data shown for untreated and 4d TGFβ treated cells). (**B**) Visualization using MetaXpress software of segmented fibronectin patches in blue (FN), stress fibers in yellow (SF), focal adhesions (FA) and nuclei in red superimposed on the original immunofluorescent pictures from (A). White boxes in images with segmented FA and nuclei outline the area magnified bellow where arrows exemplify FA points recognized by the software. (**C**) Quantification of fibronectin deposition (FN), stress fiber (SF) and focal adhesion formation (FA) per cell confirmed adequate screening windows between control (3d untreated) and 4d/5d TGFβ-treated cells. Error bars indicate the mean +/− SEM (*n* = 3). Scale bar, 50 μm in original images, 10 μm in magnified images. S, segments; B, branch points; N, nodes.

To find novel druggable targets and to dissect the molecular mechanisms underlying EMT, we have established a phenotypic, high-content microscopy screen. NMuMG cells undergo an EMT when treated with TGFβ *in vitro* [19]. During this process, epithelial, cobblestone-like clusters disintegrate upon the loss of adherens and tight junctions accompanied by major transcriptional and morphological changes. Mesenchymal cells emerge that are characterized by a spindle-shaped morphology, high expression of mesenchymal marker proteins and the ability to migrate and invade into extracellular matrix. To quantitatively monitor the process of EMT, we employed high-content immunofluorescence microscopy and computer-based image analysis. In particular, we analyzed the major cytoskeletal remodeling that occurred during this process. This included the loss of cortical actin followed by the formation of actin stress fibers (SF) and the establishment of focal adhesions (FA), two structures important for cells to migrate. In addition, we assessed fibronectin deposition (FN) to account for the upregulation of mesenchymal proteins (Figure 1A). Quantification after image segmentation showed a robust increase in these mesenchymal features of NMuMG cells with a plateau starting after 4 days of TGFβ treatment (Figure 1B, 1C). In addition, quantification of stained cell nuclei was used to account for cytotoxicity effects but also for increased cell proliferation caused by a potential inhibition of TGFβ-induced cell cycle arrest. Comparing phenotypic differences between the epithelial and mesenchymal state versus standard deviations between wells in the 384-well format revealed a robust screening readout with Z’ factors of 0.55 (+/−0.19) for focal adhesions, 0.53 (+/−0.12) for stress-fibers and 0.63 (+/−0.13) for fibronectin deposition. In comparison to this screening setup, the tracking of other well characterized EMT markers, including E-cadherin, ZO1, vimentin and SMAD, was inferior or would restrict the screen to immediate TGFBR activity related changes (Supplementary Figure S1).

As a proof of concept for our screening approach, we tested the inhibitory effects of SB-431542, a known inhibitor of TGFβ-induced EMT. SB-431542 is a selective inhibitor of TGFβ superfamily type I activin receptor-like kinase (ALK) receptors and blocks the activation of EMT directly at the receptor level after stimulation with TGFβ [20]. Quantification of focal adhesion formation, remodeling of the actin cytoskeleton to stress fibers and fibronectin deposition after TGFβ treatment in the presence of SB-431542 revealed a dose-dependent effect with an IC50 around 200 nM in all three parameters assessed. Moreover, cell numbers were increased in a dose-dependent manner depicting a higher proliferation rate of epithelial NMuMG cells than mesenchymal cells in line with the known ability of TGFβ to block cell cycle progression (Supplementary Figure S2).

### Screening for compounds blocking EMT

**Figure 2:**
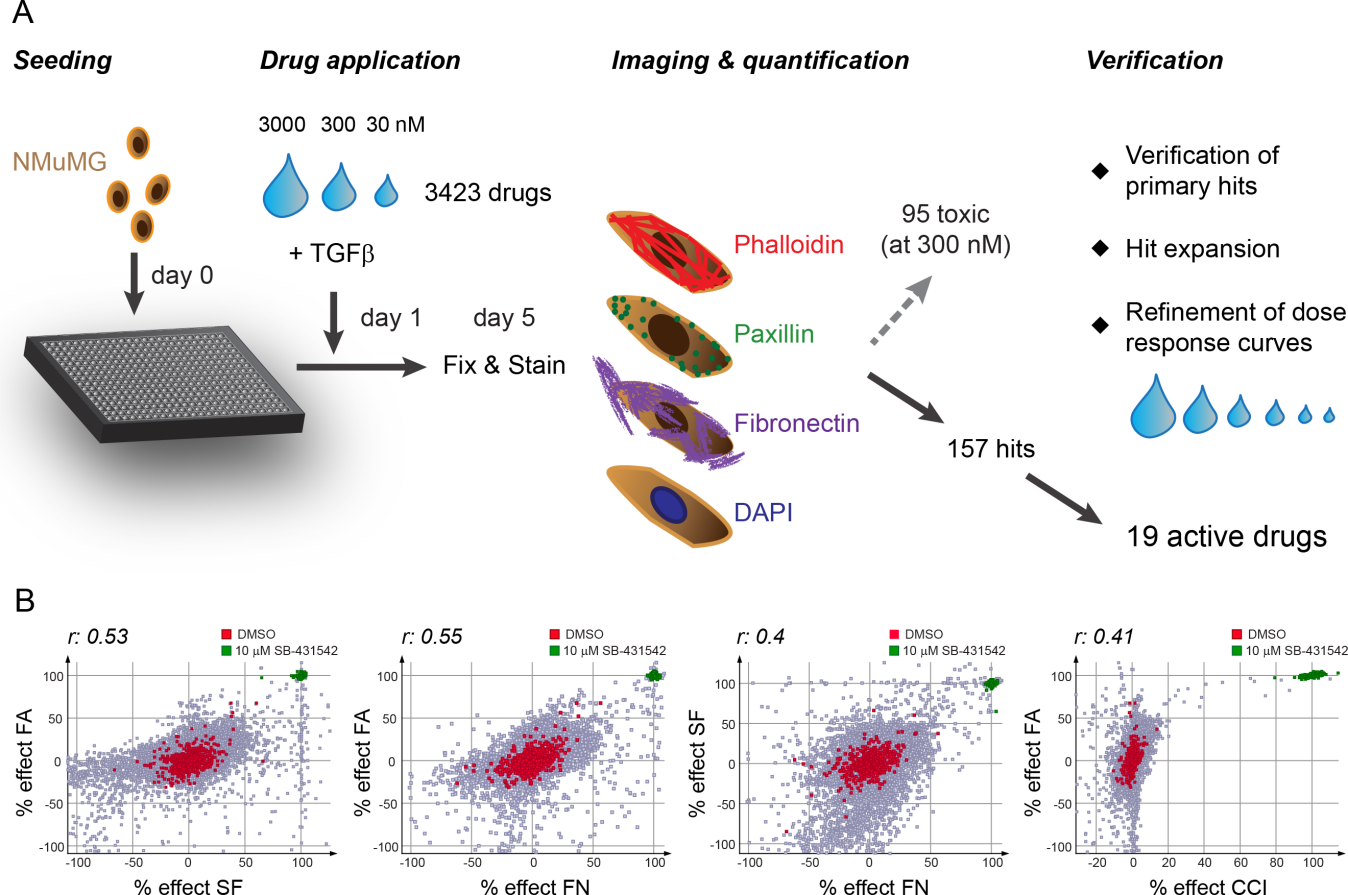
Screening procedure and correlation of screening readouts (**A**) Schematic representation of the screening procedure. One day after seeding NMuMG cells into 384-well plates, TGFβ1 (final 2 ng/ml) was added together with the compound library in a three step dilution series. After 4 days of incubation, cells were fixed and stained with phalloidin-568 to visualize filamentous actin, with paxillin antibodies for focal adhesion detection, with fibronectin antibodies for fibronectin patch formation and with DAPI to detect nuclei. The stainings were imaged, segmented and quantified using the high-content screening microscope ImageXpress and its software MetaXpress. While 95 compounds showed cytotoxicity (50% decrease in cell count compared to DMSO control) at 300 nM concentration, 157 compounds were further validated in a verification screen where the compounds were retested and dose response curves refined by using extended dilution series. At the same time, hit expansion was performed to further validate the drug targets as EMT blockers. 19 active drugs showed a robust and reproducible phenotype in all screening parameters. (**B**) Correlation of screening readouts. The comparison of the percent effects of the different screening parameters (normalized to 0% of DMSO control and 100% of 10 μM SB-431542 treatment) to each other revealed highly significant correlations between all readouts (r, Spearman correlation coefficient; *p*-value < 0.0001). FA, focal adhesions; SF, stress fibers; FN, fibronectin patches; CCI, cell count increase.

We next employed our high-content microscopy EMT screen to monitor the inhibitory effects of compounds from different libraries of approved drugs, bioactive substances and kinase inhibitors. Of the 3423 inhibitors screened, 95 compounds showed cytotoxicity as judged by at least 50% decrease in cell count at 300 nM drug concentration (Figure 2). This included drugs that were previously reported to be toxic for mesenchymal cells such as salinomycin and nigericin (Supplementary Table S1) [12]. In addition, the effect on EMT markers could not be assessed for many inhibitors against known regulators of EMT, such as EGFR and SRC, due to their cytotoxicity in the nanomolar range (Supplementary Table S1).

Comparing the inhibitory efficacies of the compound libraries on focal adhesion (FA) formation, stress fiber (SF) formation and fibronectin deposition (FN) to cell count increase (CCI), we found an adequate correlation between the screening readouts (Figure 2B). The SF readout showed the largest spread in signal compared to DMSO control in the inhibitor screen partially due to its sensitivity to compound toxicity (Figure 2B, data not shown). On the other hand, cell count was only moderately affected by most compounds, with the exception of the specific inhibitors against TGFBR, used as a positive control, providing a more stringent readout compared to SF formation (Figure 2B). Accordingly, we defined a compound as a hit in the primary screen if CCI exceeded 10% or if significant changes in FA, SF or FN pattern were detected (> 60% effect in at least one or > 40% effect in at least two pattern readouts). From our initial screen with 3423 compounds, we scored 157 hits that were verified and further validated using extended dilution series to validate the inhibitory activities of the primary hits in blocking EMT (Figure 2A, Materials and Methods). Moreover, we acquired and tested additional compounds which had published target activities comparable to the primary screening hits (compounds with asterisk in Table 1). In this secondary validation screen, we identified 19 compounds with robust and reproducible effects on all screening readouts (Table 1), while the other compounds showed varying reproducibility and cellular toxicity and were discarded. In addition to our reference compound SB-431542, we identified two other TGFBR inhibitors, RepSox [21] and SB-525334 [22] with high inhibitory efficacy on all screening readouts and an increase in cell proliferation (Table 1, Supplementary Figure S3). The SRC kinase inhibitor PP1 blocked EMT, an activity that may depend on its additional described inhibition of TGFBR1 [23, 24] (Table 1, Supplementary Figure S3). Two nucleoside analogs, idoxuridine and 5-bromo-2′-deoxyuridine, inhibited EMT potentially through perturbations in DNA replication and transcription, while cytidine analogues as well as the topoisomerase inhibitor etoposide showed high cytotoxicity (Table 1, Supplementary Table S1, Supplementary Figure S4). In addition, we identified a large family of ROCK inhibitors and different multi-kinase inhibitors as screening hits (Table 1).

**Table 1:** Active compounds blocking EMT

		FA/SF/FN	Tox	CCI	Emax
Compound name	Reported drug targets	IC50 [nM]	IC50 [nM]	IC50 [nM]	%
SB-431542	TGFβ-typeI receptors (ALK5,4,7)	243/184/195	> 25000	584	100
RepSox	TGFBR1 (ALK5)	47/55/44	> 3000	88	100
SB-525334	TGFBR1 (ALK5) and ALK4	276/243/261	> 3000	380	100
PP1	SRC family (LCK, FYN), TGBR1, (c-KIT, EGFR)	910/1136/1515	11400	374	22.3
Idoxuridine		1010/1173/948	> 25000	291	12.9
5-Bromo-2′-deoxyuridine		719/632/321	> 3000	300	19
Fasudil	ROCK1 & 2	4657/2723/1690	> 25000	2870	28.2
Y-27632 2HCl	ROCK1 & 2	2653/1313/1186	> 25000	1112	17.6
GSK429286A*	ROCK1 & 2	127/82/264	> 25000	142	33.2
Rho kinase inhibitor V*	ROCK1 & 2	425/571/374	6227	106	13.4
SR-3677*	ROCK1 & 2	160/31/179	> 25000	50.6	34.7
GSK269962*	ROCK1 & 2	2/1/7	3640	3.7	36.7
SB-772077B*	ROCK1 & 2	51/29/54	5330	50.5	30.5
Blebbistatin	Myosin II	4220/3320/2550	> 5130	n.d.	29.4
PD-161570	FGFR1, PDGFRB	337/702/1044	4970	525	50.8
PD-166285	c-SRC, FGFR1, EGFR, PDGFRB	27/79/39	229	17	24
Nintedanib	VEGFR1/2/3, FGFR1/2/3(4), PDGFRA/B, SRC family (LCK, LYN, SRC), FLT-3	2630/5060/1182	23000	1108	26.2
Sorafenib	RAF-1, BRAF, VEGFR2, FLT-3, c-KIT	2090/3926/1353	12600	103	17.5
Pazopanib	VEGFR1/2/3, FGFR, PDGFRA/B, c-KIT, c-FMS	8030/7300/3950	15700	1080	17.6

Listed are compounds that inhibit EMT progression with their name and reported drug targets according to the existing literature. IC50 values for focal adhesion (FA), stress fibers (SF), fibronectin patch (FN) formation and cell count increase (CCI) as well as the maximal effect on cell count (Emax) compared to the reference compound 10 μM SB-431542 are reported (see Materials and Methods for details). The concentration for cellular toxicity is indicated by the Tox IC50 value. Asterisks (*), compounds added during screen verification. n.d., not determined.

To test whether the hit compounds also affected other EMT-related features, we followed the two epithelial proteins E-cadherin and ZO1 as well as the mesenchymal protein vimentin by immunofluorescence staining. Upon TGFβ treatment, E-cadherin and ZO1 were internalized and downregulated resulting in the disintegration of adherens and tight junctions, respectively. In contrast, vimentin levels increased upon TGFβ addition, and vimentin-positive intermediate filaments were formed (Supplementary Figures S5 and S6). In the presence of the TGFBR inhibitor SB-431542 this transition was completely blocked: While E-cadherin and ZO1 remained at the membrane, vimentin protein formed a loose cytoplasmic meshwork and its levels were strongly reduced. Similarly, most of the hit compounds from the EMT screen reduced the dissolution of adherens and tight junctions as well as the formation of vimentin-positive intermediate filaments (Supplementary Figures S5 and S6). Although the vimentin staining decreased upon idoxuridine and sorafenib treatment, no dose-response curve could be observed indicative for non-specific effects of the drugs on this staining (data not shown). Moreover, the effects of sorafenib at concentrations that blocked EMT in the initial screen only marginally stabilized E-cadherin and ZO1 at the membrane (Supplementary Figures S5 and S6).

### ROCK pathway inhibition as main target of EMT

**Figure 3:**
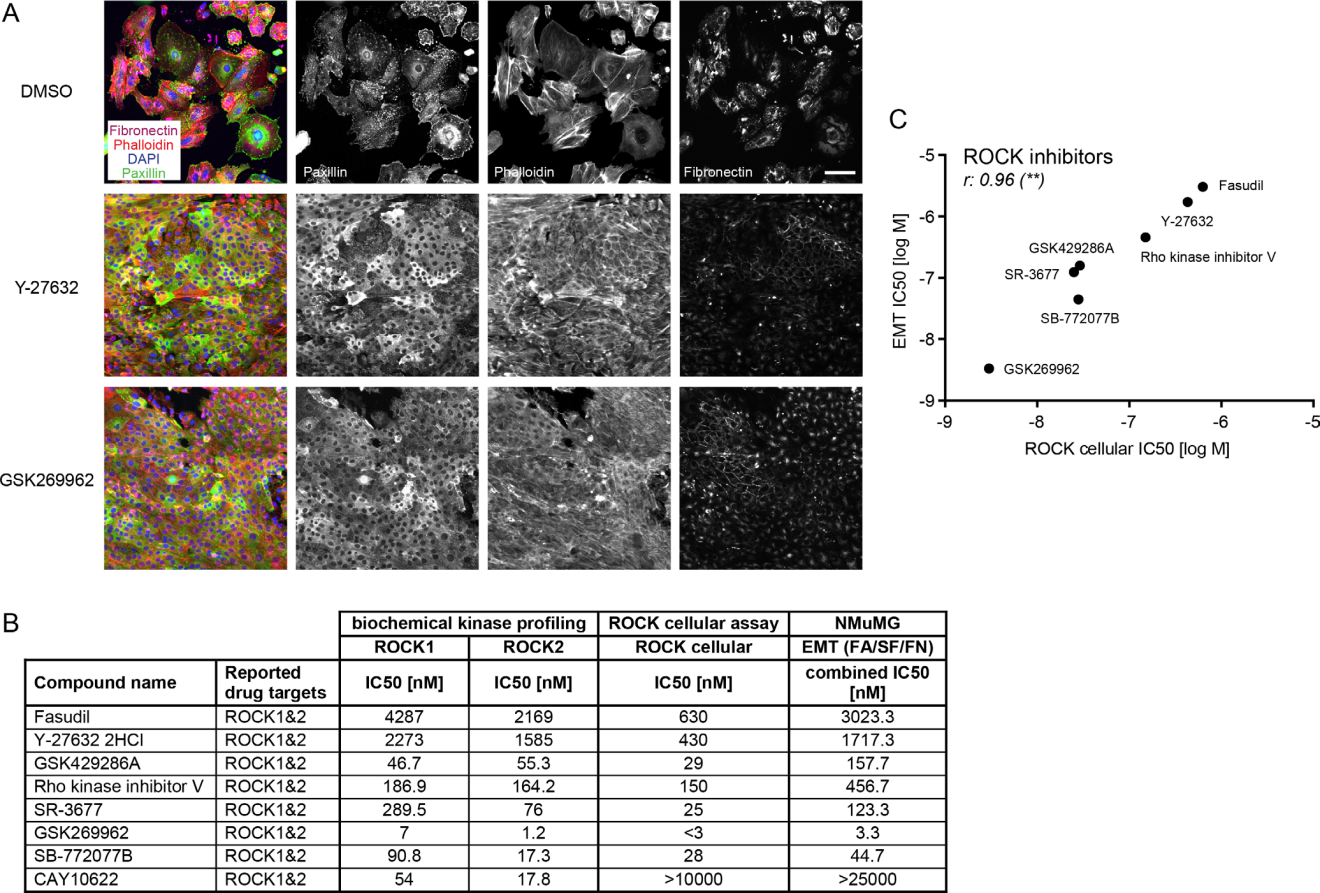
ROCK inhibitors block EMT progression (**A**) NMuMG cells were treated for four days with TGFβ and either DMSO as a control or with the ROCK inhibitors Y-27632 (8 μM) or GSK269962 (10 nM). Focal adhesions were visualized with paxillin antibodies, filamentous actin with phalloidin-568, fibronectin deposition with fibronectin antibodies and cell nuclei with DAPI. Note the reduction of fibronectin signal, of punctuated (focal adhesion) versus cytoplasmic paxillin staining and the formation of cortical actin instead of stress fibers with the ROCK inhibitors compared to DMSO control. Scale bar, 100 μm. (**B**) Pan-ROCK inhibitors and their activity on ROCK1 and ROCK2 *in vitro* (biochemical kinase profiling), on MLC phosphorylation in cells (ROCK cellular assay) and their effect on the EMT readout in NMuMG cells are shown. The combined IC50 for EMT parameters represents the mean of IC50 values obtained for FA, SF, FN patterns. (**C**) Comparing the IC50s for EMT (combined IC50 for EMT parameters FA, SF, FN) and the IC50s for the ROCK cellular assay revealed a significant correlation between the two parameters. r, Spearman correlation coefficient; **p ≤ 0.01. FA, focal adhesions; SF, stress fibers; FN, fibronectin patches.

The largest group of compounds that blocked EMT in the screen belonged to the ROCK inhibitor family (Table 1, Figure 3A, Supplementary Figure S7). This included seven ROCK inhibitors that are reported to target both ROCK1 and ROCK2 kinases. While fasudil and Y-27632 were tested in the primary screen and scored as hits, additional pan-ROCK inhibitors were subsequently tested in the secondary screen. The IC50 concentrations for EMT blocking activity based on FA, SF and FN readouts ranged between low nanomolar and micromolar concentrations. To investigate whether their effect on the screening parameters correlated with their potency in inhibiting ROCK1 and ROCK2 kinase activity, we performed *in vitro* biochemical kinase assays and assessed the phosphorylation of myosin light chain (MLC) on Thr18/Ser19 in cellular assays (Figure 3B). Phosphorylation of MLC is regulated directly by ROCK kinases and indirectly through a ROCK-dependent inhibitory phosphorylation of the MLC phosphatase [25]. Indeed, the efficiency of the ROCK inhibitors in blocking EMT closely correlated with their capability to inhibit ROCK in the cellular assay (Figure 3C). The inhibitor GSK269962 [26], targeting both ROCK1 and ROCK2 to similar extent, showed the strongest effect on EMT progression with IC50s for FA, SF, FN and CCI in the lower nanomolar range, while the pan-ROCK inhibitor CAY10622 [27] inhibited ROCK1 and ROCK2 in the biochemical kinase assay but had no effect in the cellular assay for ROCK activity nor on EMT (Table 1, Figure 3B). Fasudil and Y-27632, two widely used pan-ROCK inhibitors [28, 29], blocked the appearance of mesenchymal features in the EMT screen only in the micromolar range, in line with their reduced potency in ROCK1 and ROCK2 inhibition *in vitro* compared to the more potent inhibitor GSK269962. In agreement with ROCK's function in regulating myosin activity, blebbistatin [30], a myosin II inhibitor, showed a pronounced inhibitory effect on EMT progression (Table 1, Supplementary Figure S7). Since the activity of the ROCK inhibitors on EMT markers and cell count closely correlated with their *in vitro* biochemical and cellular activity and since none of the ROCK inhibitors showed activity towards TGFβ receptors (Supplementary Table 2), we conclude that the activation of ROCK kinases is a major pathway required for EMT.

### EMT progression is blocked by RTK inhibitors

**Figure 4:**
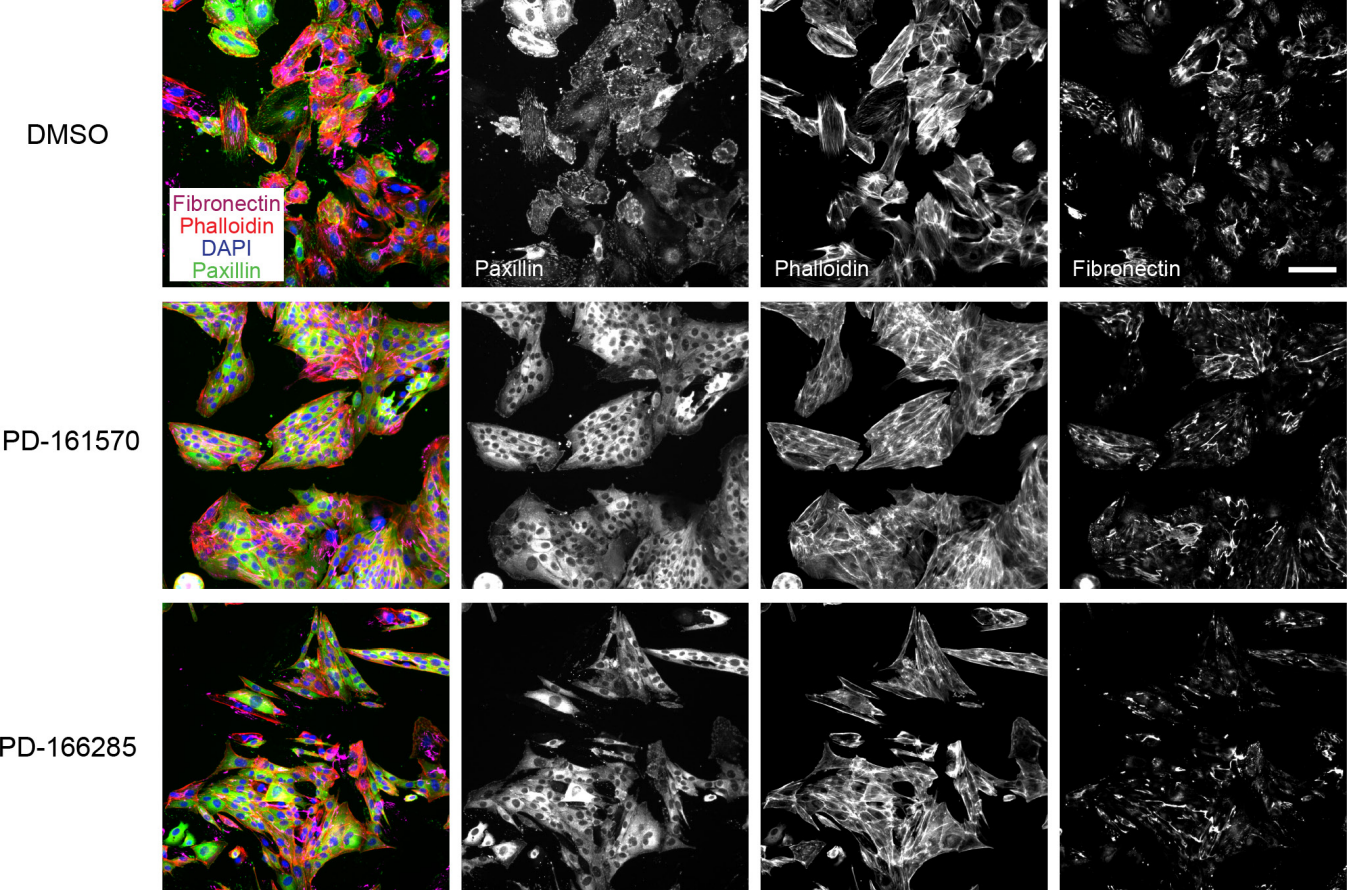
Multikinase inhibitors targeting FGFR and PDGFR block EMT progression NMuMG cells were treated and stained as described in Figure 3A. PD-166285 was used at 0.1 μM and PD-161570 at 1 μM. Scale bar, 100 μm.

Many multi receptor tyrosine kinase (RTK) inhibitors blocked EMT progression in the micromolar range, including nintedanib (BIBF-1120) [31], sorafenib [32] and pazopanib [33], while other anti-angiogenic drugs, such as vandetanib [34] and axitinib [35], were toxic to the cells (Table 1, Supplementary Table 1). Strong EMT-blocking activity was found for PD-161570 [36], an inhibitor published to target fibroblast growth factor receptor 1 (FGFR1) and to a lesser extend platelet-derived growth factor receptor beta (PDGFRB) (Table 1, Figure 4). Similarly, PD-166285 [37], a multi-kinase inhibitor with known activity against c-SRC, FGFR1, EGFR and PDGFRB efficiently repressed EMT (Table 1, Figure 4). To investigate whether FGFR inhibition or rather PDGFR inhibition would be responsible for the observed EMT blockade, we tested multiple selective FGFR or PDGFR inhibitors (Table 2). While the FGFR inhibitors failed to affect EMT at non-toxic concentrations, PDGFR inhibitors exhibited no (TSU-68 inhibitor [38]) or only partial effects on stress fiber formation and fibronectin deposition (CP673451 inhibitor [39]) (Table 2). However, CP673451 also reduced ROCK activity in a ROCK cellular assay for MLC phosphorylation, indicating that this partial effect could be mediated via ROCK inhibition (Table 2). Together, these results suggest that the strong activity against EMT progression of PD-161570 and PD-166285 is not a result of selective PDGFR or FGFR inhibition but may arise from potentially associated “off-target” effects.

**Table 2:** FGFR and PDGFR specific inhibitors only block EMT when ROCK or TGFBR activity is affected

		NMuMG				ROCK cellular assay
		FA/SF/FN	Tox	CCI	Emax	ROCK cellular
Compound name	Reported drug targets	IC50 [nM]	IC50 [nM]	IC50 [nM]	%	IC50 [nM]
PD-161570	FGFR1, PDGFRB	337/702/1044	4970	525	50.8	n.d.
PD-166285	c-SRC, FGFR1, EGFR, PDGFRB	27/79/39	229	17	24	n.d.
PD-166866	FGFR1	n.m.	3364	n.m.	n.m.	n.d.
AZD4547	FGFR1,2,3,(4), VEGFR2	n.m.	3030	n.m.	n.m.	n.d.
BGJ398	FGFR1,2,3,(4), (VEGFR2)	n.m.	1500	n.m.	n.m.	n.d.
PD-173074	FGFR1, VEGFR2	n.m.	3225	n.m.	n.m.	n.d.
CP673451	PDGFRA/B, (c-KIT, VEGFR1/2)	n.m./2560/2050	3590	n.m.	n.m.	1500
TSU-68	PDGFRB, FGFR1, VEGFR2	ia	ia	ia	1.7	ia

		biochemical kinase profiling											
		ROCK1	ROCK2	EGFR	FGFR1	FGFR2	FGFR3	FGFR4	PDGFRA	PDGFRB	SRC	TGFBR1	TGFBR2
Compound name	Reported drug targets	IC50 [nM]	IC50 [nM]	IC50 [nM]	IC50 [nM]	IC50 [nM]	IC50 [nM]	IC50 [nM]	IC50[nM]	IC50[nM]	IC50 [nM]	IC50 [nM]	IC50 [nM]
PD-161570	FGFR1, PDGFRB	ia	ia	93.4	10.4	6.5	27.2	667.2	2371	95.8	5.2	**44.9**	**1282**
PD-166285	c-SRC, FGFR1, EGFR, PDGFRB	ia	ia	6.1	4.8	1.8	9.0	55.5	51.8	2.2	< 0.3	**6.8**	**137.1**
PD-166866	FGFR1	ia	ia	ia	22.5	10.2	49.3	491.5	ia	7799	ia	**ia**	**ia**
AZD4547	FGFR1,2,3,(4), VEGFR2	ia	ia	ia	0.5	0.7	5.2	11.2	ia	1007	760.2	**ia**	**8769**
BGJ398	FGFR1,2,3,(4), (VEGFR2)	ia	ia	ia	3.2	2.6	8.2	27.4	ia	7430	7939	**ia**	**ia**
PD-173074	FGFR1, VEGFR2	ia	ia	ia	12.3	7.1	28.2	418.4	ia	3394	6733	**ia**	**ia**
CP673451	PDGFRA/B, (c-KIT, VEGFR1/2)	3373	1001	2116	5631	1007	9091	ia	617.7	1.9	1074	**ia**	**ia**
TSU-68	PDGFRB, FGFR1, VEGFR2	2062	3284	ia	2606	590.4	1473	9445	78.2	102.8	ia	**ia**	**ia**

Top list: FGFR- and PDGFR-specific compounds, their reported drug targets according to the existing literature and their effect on EMT (FA/SF/FN, CCI, Emax), cellular toxicity (Tox) and their inhibitory potential on MLC phosphorylation in cells (ROCK cellular assay) (for parameter description see Table 1 and Materials and Methods). Bottom list: FGFR- and PDGFR-specific compounds and their IC50 for inhibiting a panel of kinases *in vitro* belonging to the ROCK, EGFR, FGFR, PDGFR, SRC and TGFBR families. n.m., not measurable (no measurable effects at non-toxic concentrations); n.d., not determined; ia, inactive (> 10000 nM).

FA, focal adhesions; SF, stress fibers; FN, fibronectin patches; CCI, cell count increase.

### TGFβ receptor inhibition as main mediator for blocking EMT

**Figure 5:**
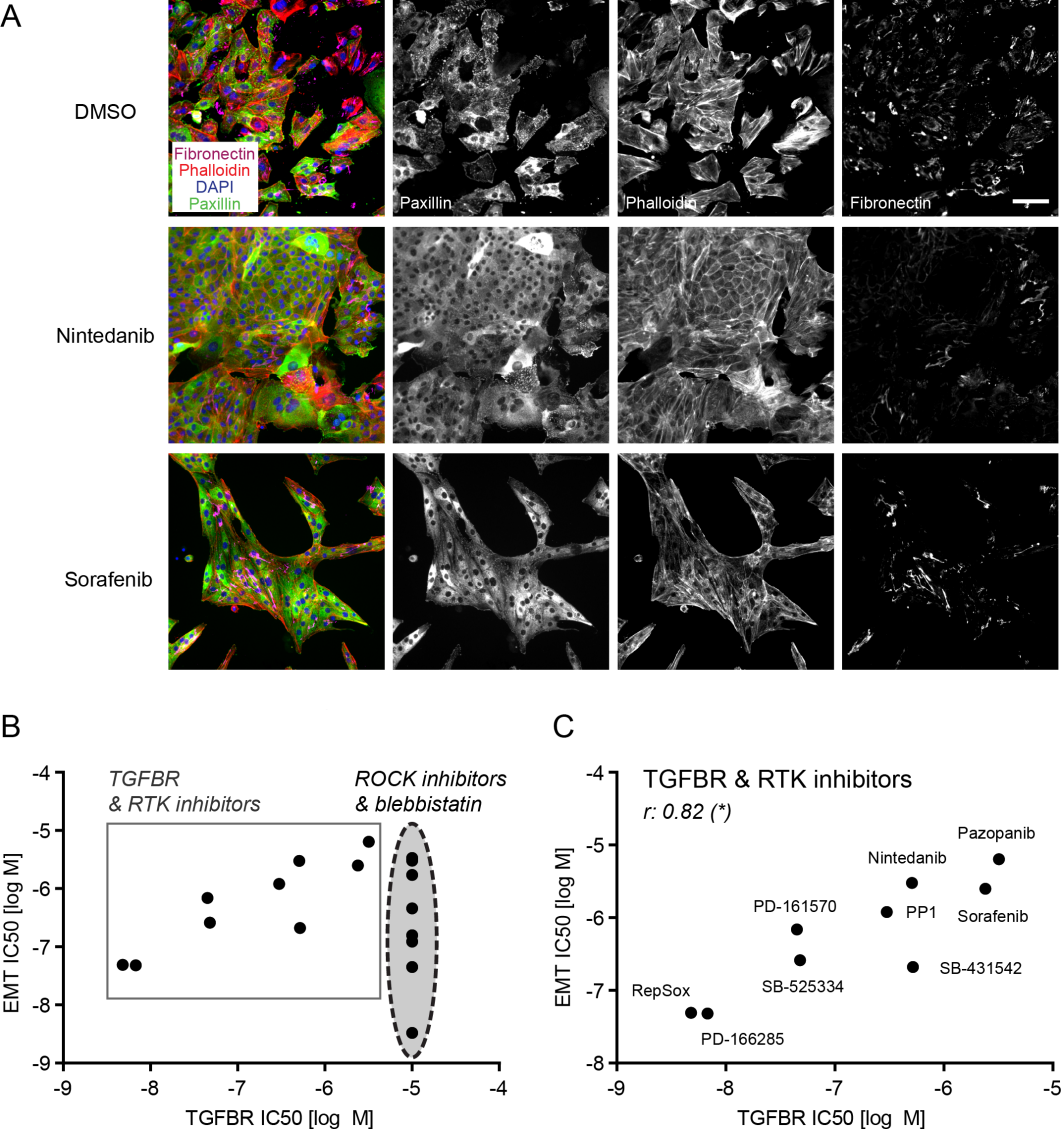
Receptor tyrosine kinase inhibitors block EMT through their “off-target” activity on TGFBR (**A**) NMuMG cells were treated and stained as described in Figure 3A. Nintedanib and sorafenib were used at 10 μM. Scale bar, 100 μm. (**B**) The comparison of IC50s for EMT (combined IC50 for EMT parameters FA, SF, FN) to the IC50s for TGFBR inhibition from the biochemical profiling of all active screening compounds (except the uridine analogues) revealed two clusters of drugs: the ROCK pathway inhibitors, which blocked EMT but did not display activity towards TGFBR, and the TGFBR and RTK inhibitors that showed inhibitory activity towards EMT in cells and towards TGFBR *in vitro*. (**C**) Comparing only TGFBR and RTK inhibitors based on their EMT IC50 and TGFBR IC50 (see B) revealed a significant correlation between the two parameters. r, Spearman correlation coefficient; *p ≤ 0.05. FA, focal adhesions; SF, stress fibers; FN, fibronectin patches.

To directly assess the kinase target selectivities of the compounds, we assessed their biochemical kinase profiles *in vitro*. The inhibitory activities of the compounds on EGFR, FGFR, PDGFR, VEGFR, SRC and TGFBR family members was compared to their efficacies in inhibiting EMT. Interestingly, the potent PDGFRB and FGFR1 inhibitor PD-161570 and PD-166285 strongly reduced the kinase activity of TGFBR1 and SRC with similar potency to their reported targets (Table 3). PP1, a SRC family inhibitor that was reported to also inhibit TGFBR1, indeed showed activity towards TGFBR1 in the biochemical kinase assay correlating with its activity in blocking EMT (Table 3) [24]. Moreover, the anti-angiogenic multi-RTK inhibitors nintedanib, pazopanib and sorafenib inhibited EMT progression and cell count increase at concentrations that substantially ranged above (up to 100-fold) their activities against their primary targets, such as receptors of the VEGFR, PDGFR or FGFR families, pointing to an “off-target” effect (Table 3, Figure 5A, Supplementary Figure S4). Indeed, nintedanib and pazopanib or sorafenib were active against TGFBR1 and TGFBR2, respectively, within a similar concentration range as their IC50 on EMT progression. Sorafenib, a multi-kinase inhibitor which in addition inhibits RAF kinase activity at low nanomolar concentrations [32], showed an effect on CCI with an IC50 in the nanomolar range (Table 1) but inhibited EMT progression in the micromolar range (Table 3), indicating that the effect of sorafenib on EMT and CCI might be caused by the repression of different molecular targets, for example TGFBR2 for affecting EMT and RAF for modulating proliferation.

**Table 3:** Receptor tyrosine kinase inhibitors directly block TGFBR kinase activity *in vitro*

		biochemical kinase profiling														NMuMG
		EGFR	FGFR1	FGFR2	FGFR3	FGFR4	c-KIT	PDGFRA	PDGFRB	VEGFR1	VEGFR2	VEGFR3	SRC	TGFBR1	TGFBR2	EMT (FA/SF/FN)
Compound name	Reported drug targets	IC50 [nM]	IC50 [nM]	IC50 [nM]	IC50 [nM]	IC50 [nM]	IC50 [nM]	IC50[nM]	IC50[nM]	IC50[nM]	IC50[nM]	IC50[nM]	IC50[nM]	IC50[nM]	IC50[nM]	combined IC50 [nM]
SB-431542	TGFβ-typeI receptors (ALK5,4,7)	ia	ia	ia	ia	ia	n.d.	ia	ia	n.d.	n.d.	n.d.	ia	**516**	**ia**	388
RepSox	TGFBR1 (ALK5)	ia	ia	4759	ia	ia	5242	ia	4373	1081	263.6	1639	6105	**4.8**	**3069**	48.7
SB-525334	TGFBR1 (ALK5) and ALK4	ia	ia	ia	ia	ia	ia	ia	ia	<0.3	3523	ia	5616	**48**	**ia**	260
PD-161570	FGFR1, PDGFRB	93.4	10.4	6.5	27.2	667.2	n.d.	2371	95.8	n.d.	n.d.	n.d.	5.2	**44.9**	**1282**	694.3
PD-166285	c-SRC, FGFR1, EGFR, PDGFRB	6.1	4.8	1.8	9	55.5	n.d.	51.8	2.2	n.d.	n.d.	n.d.	<0.3	**6.8**	**137.1**	48.3
PP1	SRC family (LCK, FYN), TGFBR1, (c-KIT, EGFR)	232.3	644	455.9	3343	621.6	n.d.	3690	132.3	n.d.	n.d.	n.d.	56.6	**296.8**	**1307**	1187
Nintedanib	VEGFR1/2/3, FGFR1/2/3/(4), PDGFRA/B, SRC family (LCK, LYN, SRC), FLT-3	ia	58.4	33.4	121.7	465.4	n.d.	20	9.5	34*	21*	13*	36.4	**505**	**ia**	2957.3
Sorafenib	RAF-1, BRAF, VEGFR2, FLT-3, c-KIT	ia	826	214.3	1389	9630	473.7	261.1	101.2	238.9	11.8	984.5	5849	**ia**	**2355**	2456.3
Pazopanib	VEGFR1/2/3, FGFR, PDGFRA/B, c-KIT, c-FMS	ia	312.8	311.3	1099	9064	120	166.8	237.3	70.7	24.2	1108	3348	**ia**	**3198**	6426.7

Listed are active compounds that inhibit receptor tyrosine kinases or have a reported activity on TGFBR. An *in vitro* biochemical kinase profile was established for these inhibitors on members of the EGFR, FGFR, c-KIT, PDGFR, VEGFR, SRC and TGFBR families and compared to their effect on EMT progression judged by the combined IC50 for FA/SF/FN. Note the inhibitory activity on TGFBR by PD-161570, PD-166285, nintedanib, sorafenib and pazopanib, all RTK inhibitors with so far unknown, direct inhibitory effects on TGFBR. Values for nintedanib marked with asterisks (*) were obtained from the literature [29]. n.d., not determined; ia, inactive (>10000 nM). FA, focal adhesions; SF, stress fibers; FN, fibronectin patches.

Comparing IC50 values for EMT blockade to the IC50 values for inhibiting biochemical *in vitro* kinase activities of TGFBR1 or 2, we could identify two different groups among our screening hits: the compounds inhibiting the ROCK pathway (direct ROCK inhibitors and blebbistatin) did not have any “off-target” effects on TGFBR and attained IC50 values for focal adhesion, stress fiber formation and fibronectin deposition that significantly correlated with their activity towards ROCK1 and ROCK2 (Figures 5B, 3C). On the other hand, inhibitors reported to target TGFBR, such as SB-431542, RepSox, SB-525334 and PP1, together with the multi-kinase inhibitors PD-161570, PD-166285, nintedanib, pazopanib and sorafenib revealed a significant correlation between their efficacy in blocking EMT and their inhibition of TGFBR1 or 2 (Figure 5C).

Based on these results, we conclude that the EMT blocking effect observed by receptor tyrosine kinase inhibitors most likely arises from their “off-target” activity towards TGFBR rather than from their activities against other kinases including RTKs of the FGFR, PDGFR or VEGFR families.

### SMAD phosphorylation decreases in NMuMG cells upon treatment with inhibitors that biochemically block TGFBR

**Figure 6:**
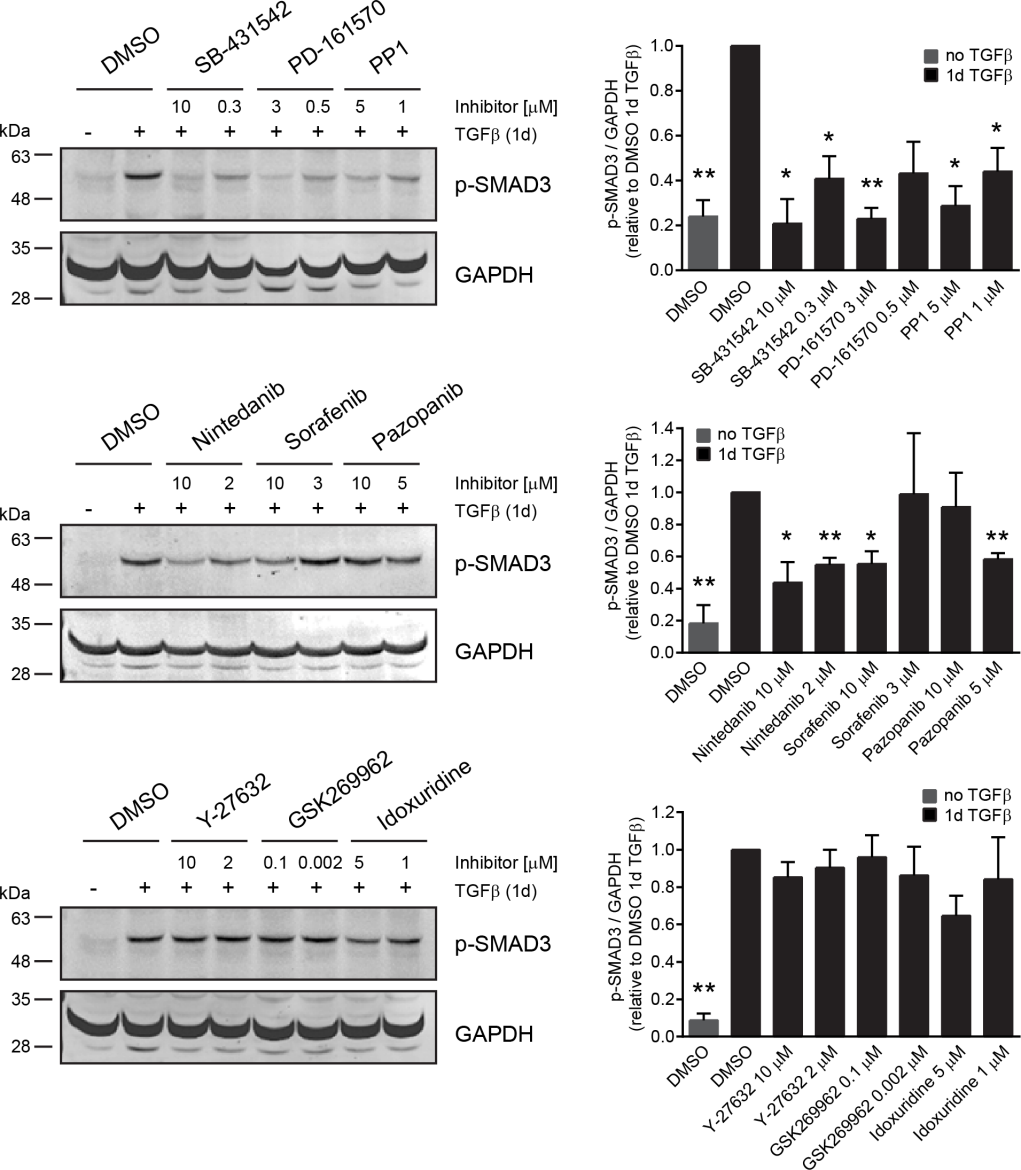
Receptor tyrosine kinase inhibitors but not ROCK inhibitors block SMAD phosphorylation in cells NMuMG cells were treated for one day with TGFβ and either DMSO as a control or with a panel of inhibitors representing the different groups of EMT hits. As an epithelial control, cells were treated for one day with DMSO in the absence of TGFβ. Every inhibitor was tested at two different concentrations, either close to the maximal effect concentration or to the EMT IC50 from the screen. SMAD3 phosphorylation was followed and quantified against GAPDH using immunoblotting. Error bars indicate the mean +/− SEM (*n* = 3). Significant changes of p-SMAD3/GAPDH ratio between DMSO control after 1d of TGFβ treatment and inhibitor with TGFβ or DMSO control in the absence of TGFβ were determined using paired *t*-tests. */**p ≤ 0.05/0.01. Note that pazopanib affects SMAD phosphorylation only at concentrations below 10 μM due to increased toxicity of the drug at the maximum concentration.

To test whether the inhibitors which repress TGFBR activity *in vitro* would also affect TGFBR signaling in cells, we treated NMuMG cells for one day with TGFβ in the presence of DMSO or one of the inhibitors of our screening hits (Figure 6). To determine TGFBR activity we quantified the phosphorylation of a direct TGFBR target, SMAD3, by immunoblotting and LI-COR analysis. Without TGFβ stimulation, SMAD3 phosphorylation was nearly absent. Similarly, the co-treatment of TGFβ with SB-431542 blocked SMAD3 phosphorylation, both at high inhibitor concentrations as well as at the IC50 concentration for EMT inhibition. All compounds with activity against TGFBR in the biochemical kinase assays also significantly decreased SMAD3 phosphorylation in NMuMG cells (Figure 6, Table 3). In contrast, SMAD3 phosphorylation remained unchanged by treatment with inhibitors that did not to block TGFBR activity, such as the ROCK inhibitors, Y-27632 and GSK269962, and idoxuridine (Figure 6, Supplementary Table S2).

Taken together, these results show that the “off-target” activity on TGFBR by different multi-kinase inhibitors substantially affects TGFBR signaling in NMuMG cells and consequently directly blocks TGFβ-induced EMT.

### ROCK inhibitors block EMT in murine breast cancer cells

**Figure 7:**
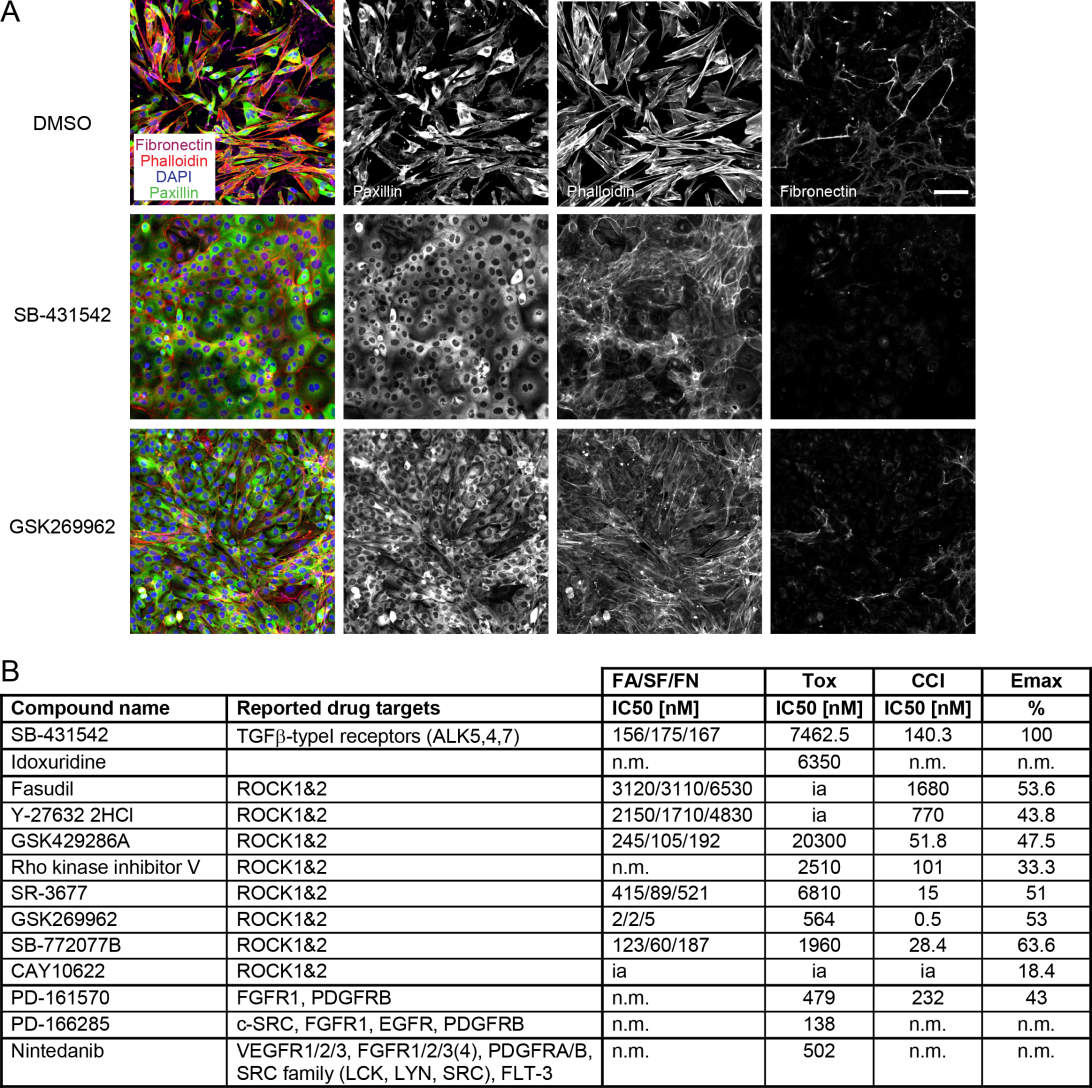
TGFβ-induced EMT in Py2T cells is blocked by ROCK inhibitors (**A**) Py2T cells were treated and stained as described in Figure 3A. SB-431542 was used at 10 μM and GSK269962 at 10 nM. Scale bar, 100 μm. (**B**) A selection of EMT-blocking compounds from the EMT screen in NMuMG cells (see Table 1) were retested for their ability to block EMT in TGFβ-treated Py2T cells. Listed are the names and reported drug targets of the compounds according to existing literature. IC50 values for FA, SF, FN and cell count increase (CCI) as well as the maximal effect on cell count (Emax) compared to the reference compound 10 μM SB-431542 are reported (see Materials and Methods for details). The concentration for cellular toxicity is indicated by the Tox IC50 value. n.m., not measurable (no measurable effects at non-toxic concentrations); ia, inactive (> 25000 nM). FA, focal adhesions; SF, stress fibers; FN, fibronectin patches.

Since NMuMG cells are untransformed, normal mammary epithelial cells, we validated our screening hits in a breast cancer cell line. To this end, we tested SB-431542, idoxuridine, several ROCK inhibitors, the inhibitors PD-161570 and PD-166285 that showed a potent “off-target” effect on TGFBR (Table 2) and the multi-kinase inhibitor nintedanib on murine Py2T breast cancer cells. Py2T cells have been derived from mammary tumors of the MMTV-PyMT mouse model of breast cancer and undergo a TGFβ-dependent EMT *in vitro* and *in vivo* [40]. Upon the addition of TGFβ, epithelial Py2T cells underwent a similar cytoskeletal rearrangement as NMuMG cells, including the formation of focal adhesions and actin stress fibers (Figure 7A). At the same time, they increased their fibronectin production and deposition (Figure 7A). Treatment of Py2T cells with the TGFBR inhibitor SB-431542 largely blocked TGFβ-induced EMT leading to a reduction of EMT markers with an IC50 around 200 nM and the maintenance of epithelial cell morphology (Figure 7). Similarly, the formation of focal adhesions, stress fibers and fibronectin deposition was reduced with different pan-ROCK inhibitors (Figure 7, Supplementary Figure S8). Importantly, the inhibitory effect of ROCK inhibitors largely correlated with their efficacy in blocking EMT in NMuMG cells as well as with their potency in inhibiting ROCK in different *in vitro* assays (Figure 7B, Figure 3B). Accordingly, GSK269962 potently inhibited the cytoskeletal rearrangement and fibronectin deposition in the lower nanomolar range. Another pan-ROCK inhibitor, CAY10622, did not affect EMT progression in Py2T cells nor in NMuMG cells or in the cellular ROCK assay (Figure 7, Figure 3). The effect of idoxuridine, PD-161570, PD-166285 and nintedanib on EMT inhibition in Py2T cells could not be validated since they exhibited increased cytotoxicity in these cells (Figure 7B, Table 1).

Together, the data indicate that ROCK signaling is important for a TGFβ-induced EMT not only in normal mammary epithelial cells but also in murine breast cancer cells.

## DISCUSSION

Recent studies in mouse models and the characterization of human breast cancer subtypes have further solidified the involvement of EMT in the metastatic cascade, especially under chemotherapy treatment, and as a potential predictor for inferior response to breast cancer therapy [3, 6, 7]. Interfering with EMT and its associated gain in cell migration, invasion and cancer stem cell properties might represent an important therapeutic strategy in the treatment of the metastatic disease. To find pharmacological modulators and druggable targets during EMT, we have established a high-content microscopy-based screen. Combining different parameters of EMT progression such as remodeling of cortical actin to stress fibers, the formation of focal adhesions, the upregulation of the mesenchymal protein fibronectin and TGFβ-induced cell cycle arrest, we have screened 3423 compounds with assigned pharmacological activities for their potential to inhibit EMT. The feasibility and robustness of the screening setup has been confirmed by Z’ factors above 0.5 for all screening readouts and by the identification of multiple TGFβ receptor inhibitors from the compound library as screening hits.

The largest subset of screening hits are ROCK inhibitors (Table 1), underlining the importance of cytoskeletal rearrangements during EMT for cells to elongate and to gain directed motility [11]. Small RHO GTPases such as RHOA, RAC1 and CDC42 coordinate the remodeling of the cytoskeleton in space and time [25]. While inhibition of RHOA activity upon TGFβ-induced EMT leads to local disassembly of actin cytoskeleton and tight junctions, an increase of RHOA activity induces, through its downstream effector kinases ROCK, the formation of actin stress fibers and actomyosin contractility [41, 42]. ROCK phosphorylation of myosin-light chain (MLC) and inactivation of myosin-light chain phosphatase promotes actomyosin contractility, while the stimulation of LIM domain kinase (LIMK), and consequently the phosphorylation and inactivation of the actin-severing protein cofilin, stabilizes actin stress fibers [25]. Dominant-negative RHOA (N19-RHOA) or a kinase-dead ROCK construct (KD-IA p160ROCK) have been shown to block EMT progression in NMuMG cells [41]. In agreement with these results, we have identified multiple compounds targeting ROCK kinase activity with our screening approach (Table 1). The compounds’ inhibitory potential on ROCK activity in *in vitro* biochemical assays as well as in cellular ROCK assays correlate tightly with their ability to block EMT progression in NMuMG as well as in Py2T breast cancer cells (Figure 3, Figure 7). Fasudil, which has potent vasodilatory effects and is approved in Japan for the treatment of subarachnoid hemorrhage, and the experimentally used ROCK inhibitor Y-27632 block the cytoskeletal remodeling and fibronectin deposition upon TGFβ-treatment though only in the micromolar range due to their higher IC50 values for ROCK inhibition (Figure 3, Figure 7). The pan-ROCK inhibitor GSK269962, on the other hand, shows high potency in inhibiting ROCK kinase activity *in vitro* and in cells and blocks EMT at low nanomolar concentrations (Figure 3, Figure 7). Since both fasudil and Y-27632 have a broad inhibition spectrum and target other kinases, such as PRK2 [43], and since they are used at relatively high doses, reported treatment effects *in vitro* and *in vivo* may not solely arise from ROCK inhibition. Nevertheless, studies in mice predict a beneficial role of ROCK inhibition in treatment of cancer. For instance, Y-27632 treatment reduces bone metastasis in the SUM1315 breast cancer xenograft mouse model [44]. Similarly, blocking ROCK activity in mice with myeloproliferative disease significantly prolongs their lifespan due to enhanced apoptosis and reduced growth of leukemic cells [45]. Therefore, in light of the potential benefits of ROCK inhibition also in cancer therapy, it will be interesting to see whether the less studied yet more selective and more potent ROCK inhibitors will show advantages in blocking tumor progression and metastasis in mouse models.

While inhibition of EGFR and SRC results in cytotoxicity during the EMT process, we have identified multiple receptor tyrosine kinase inhibitors known to target members of the FGFR, PDGFR and VEGFR families to interfere with EMT (Table 1, Supplementary Table S1). Previous studies have reported that FGF, PDGF and VEGF can exert a similar role as HGF, EGF and TGFβ in the induction of EMT [1]. Moreover, FGF, PDGF and VEGF receptors are upregulated in their expression during TGFβ-induced EMT [46–48]. The induction of PDGF or VEGF receptor signaling as a consequence of EMT has been shown to promote vessel functionality and angiogenesis in murine breast tumor models, but may not be directly required for TGFβ-induced EMT [47, 48]. Accordingly, we have not been able to reproduce the potent EMT inhibiting effects of PD-161570 and PD-166285, primary hits in our screen, with alternative inhibitors targeting either FGFR or PDGFR. While the PDGFR inhibitor TSU-68 has no effect on EMT, the PDGFR inhibitor CP673451 has a partial effect on stress fiber formation and fibronectin deposition, yet also exhibits “off-target” activity on ROCK kinases (Table 2). Comparably, more selective FGFR inhibitors have not blocked EMT below toxic concentrations (Table 2). Activated FGFR can phosphorylate phospholipase C and FGFR substrate 2 (FRS2), the latter providing a platform for RAS/MAPK and PI3K/AKT signaling required for cell proliferation and survival. Accordingly, inhibition of FGFR in breast cancer cells results in cell cycle arrest and induction of apoptosis [49, 50]. We speculate that increased cytotoxicity with more selective FGFR inhibitors in NMuMG cells may be due to decreased RAS/MAPK and PI3K/AKT signaling without affecting EMT at lower concentrations.

Surprisingly, biochemical kinase profiling reveals a strong inhibitory effect of PD-161570 and PD-166285 on TGFBR1 and to a lesser extent on TGFBR2 activities, which correlates with their potency in blocking EMT (Table 3, Figure 5). Interestingly, other multi-kinase inhibitors, such as nintedanib, pazopanib and sorafenib, similarly inhibit EMT in accordance with a reduction in the biochemical activity of TGFBR and a decrease in SMAD3 phosphorylation in cells (Table 3 and Figure 6). While the IC50 for *in vitro* inhibition of TGFBR2 with sorafenib lies in the micromolar range, similar to the IC50 for EMT marker inhibition in cells, the antagonistic effect of sorafenib on TGFβ-induced cell cycle arrest already occurs in the nanomolar range. The enhancing effect on proliferation may be mediated by a combination of blocking TGFβ-induced cell cycle arrest through TGFBR inhibition and of directly affecting cell proliferation by the inhibition of RAF activity. In cells with wild-type *B-Raf*, such as NMuMG cells, inhibition of RAF kinase can enhance proliferation and tumor growth *in vivo,* in contrast to mutant *B-Raf* cancer cells where RAF inhibition usually induces anti-proliferative effects [51].

Both sorafenib (Nexavar^®^) and pazopanib (Votrient^®^) are currently used in the clinics as anti-angiogenic therapy for various cancer types including renal cell carcinoma [52]. Since pazopanib's active fraction is substantially reduced by high plasma protein binding ranging above 99.9% [53, 54], pazopanib requires steady state blood levels of at least 40 μM to be active in patients. With an IC50 of 3.2 μM on TGFBR2 in biochemical assay and 6.4 μM for blocking EMT in NMuMG cells (Table 3), it is thus unlikely that inhibition of TGFBR2 and consequently EMT has any impact on pazopanib's mode of action in patients. Similarly, sorafenib reaches high drug concentrations in the 10 μM range in patients but also shows a high plasma protein binding of 99.7% [55, 56]. Whereas sorafenib's clinical activity might be limited due to its pharmacokinetic properties, preclinical *in vitro* studies with cell lines use sorafenib between 1 and 10 μM. In this range, sorafenib blocks TGFβ-mediated EMT in mouse hepatocytes in a dose-dependent manner [57]. This result is in agreement with the effect on EMT and SMAD3 phosphorylation that we observe in NMuMG cells and with a potential direct effect of sorafenib through TGFBR2 inhibition (Table 3, Figure 6). However, drug levels sufficient to inhibit TGFBR kinase may not be reached *in vivo*. Therefore EMT inhibition might not contribute to the antitumor effect of sorafenib in patients [58].

Along the same lines, nintedanib reaches only low maximum plasma concentration (C_max_) in human patients in addition to high plasma protein binding and fast clearance [52, 59]. However, cellular trapping of the drug and the generation of an active metabolite might beneficially contribute to the good efficacy of nintedanib observed *in vivo* [31]. In mice, C_max_ was reported to be around 1 μM after single dose of 50 mg/kg nintedanib [31]. Since nintedanib blocks EMT progression in NMuMG cells with an IC50 in the lower micromolar range and is able to block TGFBR1 activity in biochemical assays in the submicromolar range, it is plausible that TGFBR inhibition contributes to its beneficial effects *in vivo*. Nintedanib has shown promising results in large clinical trials for the treatment against idiopathic pulmonary fibrosis (IPF) [60] and in lung cancer patients with advanced adenocarcinoma after first-line chemotherapy [61] and has been clinically approved for these indications (as Ofev^®^ and Vargatef^®^, respectively) [52]. Interestingly, TGFβ is believed to play an important role in the pathogenesis of IPF [62]. Studies on nintedanib's mode of action in the treatment of IPF revealed, among others, an inhibitory effect on the TGFβ-stimulated differentiation of fibroblasts to myofibroblasts as well as on TGFβ-induced collagen secretion and deposition in cells derived from IPF patients [63, 64]. These data suggest that inhibition of TGFβ-signaling contributes to the therapeutic efficacy of nintedanib in IPF patients, either indirectly through c-ABL and/or ERK, as hypothesized by the authors, or directly through TGFBR inhibition, as suggested by our findings. Similarly, in agreement with nintedanib's inhibitory effect on TGFBR1 activity as well as SMAD phosphorylation and EMT in our analyses, multiple studies report a lack of EMT induction after nintedanib treatment in cancer cell lines *in vitro* and in mouse models of cancer *in vivo*. A recent study using ovarian, lung, bladder and pancreatic cancer cell lines has shown a decreased mesenchymal phenotype of these cell lines as well as of SKOV-3 xenografts after nintedanib treatment [65]. Additionally, in lung and pancreatic cancer models, nintedanib treatment has a potent anti-angiogenic effect but does not induce EMT, although tumor hypoxia, a well-described EMT inducer, is high [66]. Also, A549 lung cancer cell xenografts appear more epithelial after nintedanib treatment as compared to untreated controls [66]. Finally, unlike other anti-angiogenic treatments, there is currently no indication that nintedanib treatment would induce an EMT switch allowing cells to become more invasive and thus favoring the formation of metastasis [67]. In contrast, a recent study of the therapeutic effects of nintedanib in the RipTag2 mouse model of pancreatic β-cell carcinogenesis revealed a profound block in angiogenesis and subsequent tumor progression without increasing invasion or metastasis formation in these mice [68]. The results indicate that partial TGFBR inhibition could contribute to the beneficial therapeutic effect of nintedanib in the treatment of IPF or different tumor models.

In conclusion, we have established and validated a high-content microscopy screen for the identification of pharmacological inhibitors of EMT. Besides the high-throughput screening of pharmacological compounds and biologicals, the screening set-up may also be useful for a variety of other applications, including RNAi-mediated screening for critical players of EMT and tumor metastasis. As a validation of the screening setup, our limited screen with chemical compounds has identified a number of interesting hits, among which ROCK inhibitors certainly motivate follow-up studies. On the other hand, a number of frequently used multi-kinase inhibitors also repress TGFBR signaling and, thus, have been identified to interfere with TGFβ-induced EMT. Whether TGFβ signaling is indeed affected by these multi-kinase inhibitors in patients and whether their “off-target” effect provides additional benefits compared to other anti-angiogenic therapies warrants further investigations.

## MATERIALS AND METHODS

### Antibodies and reagents

The mouse anti-paxillin antibody was purchased from BD Biosciences (610052) and the corresponding secondary Alexa488 anti-mouse antibody from ThermoFisher Scientific (A-11029). The rabbit anti-fibronectin antibody was obtained from Sigma (F3648) and the corresponding secondary Alexa647 anti-rabbit antibody from ThermoFisher Scientific (A-21245). Alexa Fluor 568-coupled phalloidin was from ThermoFisher Scientific (A12380). Rat anti-E-cadherin antibody was obtained from ThermoFisher Scientific (13-1900), rabbit anti-ZO1 antibody from ThermoFisher Scientific (61-7300), mouse anti-vimentin antibody from Sigma (V2258), mouse anti-SMAD2/3 from BD Biosciences (610842), rabbit anti-p-SMAD3 from Cell Signaling (p-Ser423/425; 9520) and mouse anti-GAPDH from Sigma (G8795). For quantitative immunoblot analysis, secondary antibodies from LI-COR Biosciences, IRDye 680RD Goat anti-Mouse (926-68070) and IRDye 800CW Goat anti-Rabbit (926-32211) were used. DAPI was acquired from Sigma (D9542), recombinant human TGFβ1 protein from R & D Systems (240B-0-10), 16% paraformaldehyd (PFA) from Electron Microscopy Services (15710-S), fatty-acid free BSA from Calbiochem (126575), Trypsin/EDTA from Sigma (T4174), PBS from Gibco (14200-067), and Triton X-100 from Sigma (X-100).

### Chemical compound libraries

The following bioactive chemical compound libraries were screened for EMT blockade in TGFβ-treated NMuMG cells (*n* = 1 at 3000 nM, 300 nM and 30 nM): 1) The *FDA approved drug library* with 640 substances from ENZO Life Sciences (BML-2841), 2) The *LOPAC library* of 1280 pharmacologically active compounds from Sigma (LO1280), 3) the *ICCB Known Bioactives library* with 480 substances from ENZO Life Sciences (BML-2840), 4) The *Kinase Inhibitor library* with 80 substances from ENZO Life Sciences (BML-2832), and 5) 943 substances from Actelion drug discovery programs with known target specificity. All libraries were provided as 2 mM stocks dissolved in 100% DMSO. All primary hits from these libraries were verified in a larger dilution series (3000 nM, 1000 nM, 300 nM, 100 nM, 30 nM) on TGFβ-treated NMuMG cells.

### Drugs

Defined compounds used for validation experiments were ordered *de novo* from various suppliers: SB-431542, PD-173074, U0126, Salinomycin, Nigericin, PD-161570, PD-166285, PD-166866, Idoxuridine and 5-Azacytidine (all from Sigma); Y-27632 2HCl, GSK429286A, Nintedanib, Pazopanib, Sorafenib, Vatalanib, Axitinib, Sunitinib, Erlotinib, Lapatinib, Gefitinib, Dasatinib, Crizotinib, Vandetanib, AEE788, Cediranib, AZD4547, CP673451, TSU-68 and KX2-391 (all from Selleck Chemicals); PP1, PP2, Rho kinase inhibitor V, SR-3677, GSK269962, SB-772077B (all from Tocris); Fasudil from Enamine; CAY10622 and PD-166326 from Cayman; BGJ398 from Axon Medchem; 5-Aza-2′-deoxycytidine from Chem-Impex. The reordered compounds were tested in extended dilution series ranging from 25 μM to 0.04 nM on TGFβ-treated NMuMG cells and from 25 μM to 1 nM on TGFβ-treated Py2T cells.

### Cell culture

NMuMG cells (the subclone NMuMG/E9 as described by [19]) and Py2T cells [40] were grown in Dulbecco's Modified Eagle's Medium (DMEM high glucose, Sigma, D5671) supplementary with 10% fetal bovine serum (Sigma, F7524), 2 mM GlutaMax (Gibco, 35050), 100 μg/ml penicillin-streptomycin (Sigma, P4333) at 37°C and 5% CO_2_ in a humidified incubator.

### Screening: Cellular assay and staining

NMuMG and Py2T cells were trypsinized, washed, seeded at 1000 cells/well, resuspended in 40 μl growth medium with a Multidrop-384 (Titertek) in 384-well microplates (Greiner, 781091), and grown overnight at 37°C, 5% CO_2_. The next day, 111 μl growth medium was added with the Multidrop-384 to 1 μl of the 2 mM library compounds of which 10 μl was transferred with a 384-well pipettor (VPrep, Agilent, US) to the cell assay plate and equilibrated for 10 min (final compound concentration 3 μM). For screenings at 300 nM or 30 nM, the compounds were diluted 1:10 or 1:100 in growth medium, respectively, before use. Afterwards, 10 μl of 6×TGFβ in growth medium was added to the cell assay plate with the VPrep (final 2 ng/ml concentration), a 3× 20 μl mixing step was performed and cells were cultivated for 4 days at 37°C, 5% CO_2_. On every assay plate a concentration series of the reference compound SB-431542 was added to assess assay reproducibility. After 4 days, cells were fixed for 20 min in 40 μl/well 4% PFA at room temperature, washed 3× in 1× PBS with a Biotek ELX405 cell washer and permeabilized in 0.5% Triton X-100/PBS for 10 min. Subsequently, cells were washed 2× in 0.01% Triton X-100/PBS (PBS-T) and then blocked for 60 min with 1% BSA in PBS-T. Incubation with primary antibodies against paxillin (1:200) and fibronectin (1:400) in 1% BSA/PBS-T for 60 min was followed by incubation with the Alexa-Fluor coupled antibodies Alexa488 anti-mouse (1:1000) and Alexa647 anti-rabbit (1:1000) for 60 min. Together with secondary antibodies, Alexa568-coupled phalloidin (1:200) and 1 μg/ml DAPI staining was performed for 60 min. Alternatively, primary antibodies against E-cadherin (1:2000), SMAD2/3 (1:400), vimentin (1:200) or ZO1 (1:100) were used in combination with Alexa-Fluor coupled secondary antibodies. Thereafter, cells were washed with PBS-T and stored in PBS until imaging.

### Screening: Image acquisition and segmentation

Cells were imaged using the automated epifluorescence microscope ImageXpress Micro from Molecular Devices (Sunnyvale CA, USA) equipped with a plate-loading robot (CRS Catalyst, Thermo Scientific). Images were acquired from 9 sites per well using DAPI (nuclei), FITC (paxillin, FA), TxRed (phalloidin, SF) and Cy5 (fibronectin, FN) filter/dichroic combinations at 10x magnification. Alternatively, images were acquired using DAPI, FITC (E-cadherin) and TxRed (Smad2/3) or DAPI, FITC (ZO1) and TxRed (Vimentin) filter/dichroic combinations at 10x magnification. The laser autofocus was used to control axial focus of the image acquisition. The images were automatically analyzed by MetaXpress and segmented into sub-cellular structures based on morphological features such as object size and fluorescence distribution. Fluorescence distribution was a measure of the threshold intensity above local background and was adjusted for all parameters (nuclei, FA, SF and FN) by means of the respective positive and negative controls to compensate for inter-experimental variations between the different screening batches. The nuclei and FA were segmented with the Transfluor module of the MetaXpress software. The size parameter to segment the nuclei included objects between 9 and 16 μm. The FA were segmented using the parameter “vesicles” with a size between 1–10 μm. The output data used for further data analysis were the average nuclear count and the vesicle number (FA) per cell for each well. SF and FN were quantified with the Angiogenesis module of MetaXpress and the size distribution was set to 1–5 μm for both. The output data segments, branch points and nodes were summed up and divided by the number of nuclei to calculate SF and FN per cell. Vimentin was quantified similar to SF and FN using the Angiogenesis module. Smad2/3 translocation was quantified with the Translocation Module of MetaXpress. The signal-to-background (S/B) ratios for E-cadherin and ZO1 were determined from line scans through 10 representative images of epithelial NMuMG cells in the absence of TGFβ. Min and max gray levels were calculated from each line scan. The max/min ratios were calculated and the average ratio defined as S/B.

### Screening: Calculation of screening parameters

Data were imported in 384-well layout to HTS-Studio, an Actelion in-house developed data analysis and image visualization pipeline software. Data were normalized to wells that received TGFβ and vehicle (mesenchymal phenotype = 0% effect, negative control) and wells that received TGFβ and 10 μM SB-431541 (epithelial phenotype = 100% effect, positive control). Z’ factor was calculated from the mean (μ) and standard deviations (σ) of 4 positive (p) and 16 negative (n) control wells as follows: 1 – [3*(σ_p_+ σ_n_) / (μ_p_-μ_n_)]. The IC50 of the FA/SF/FN readouts depicted the concentration at which the parameters were decreased by 50% while the IC50 for cell count increase (CCI) was defined as the concentration at which cell count was increased by 50% compared to 100% with 10 μM SB-431542. The toxicity of the drugs (Tox IC50), on the other hand, was determined by calculating the concentration at which cell count is decreased by 50% compared to DMSO control and with zero cells corresponding to 100% toxicity. For calculation of IC50 values reduced data were imported into IC50-Witch, an Actelion in-house developed software for IC50 curve fitting. IC50 values were calculated according to a four-parameter logistic curve model with a variable slope. The effect of library compounds on cell count (CCI Emax) was calculated either at the tested concentrations (3000 nM, 300 nM and 30 nM in the screening) or, in the case of compound dilution series for re-ordered compounds, the highest value from any of the tested concentration was calculated. The Emax value was normalized to the negative control (0% effect) and the positive control with 10 μM SB-431542 (100% effect). The values calculated represent the average from the different experiments performed with the screening libraries and reordered compounds (see above).

### Image processing and statistical analysis

Images for figures were processed using ImageJ software and assembled in Adobe Illustrator. Signal intensities for paxillin, phalloidin-568 and DAPI stainings were adjusted individually from image to image to best visualize the structures. Fibronectin stainings of different drugs were processed together with their respective DMSO control from the same screening plate to accurately report potential changes in signal intensities. Pictures with segmented features superimposed on original immunofluorescent stainings were directly copied from MetaXpress software and adjusted in ImageJ for best visualization.

All statistical analysis was performed with GraphPad Prism software. Spearman correlations and associated two-tailed *p*-values were calculated.

### Biochemical kinase profiling

A radiometric protein kinase assay (^33^PanQinase^®^ Activity Assay) was used for measuring the kinase activity of the 16 protein kinases at ProQinase (Freiburg, Germany). All protein kinases provided by ProQinase were expressed in Sf9 insect cells or in E. coli as recombinant GST-fusion or His-tagged proteins. All kinase assays were performed in 96-well FlashPlates^™^ from Perkin Elmer in a 50 μl reaction volume. The assay for all protein kinases contained 70 mM HEPES-NaOH pH 7.5, 3 mM MgCl_2_, 3 mM MnCl_2_, 3 μM Na-orthovanadate, 1.2 mM DTT, 50 μg/ml PEG20000, ATP (variable concentrations, corresponding to the apparent ATP-Km of the respective kinase), [γ-33P]-ATP, protein kinase, test compound, and substrate. All compounds were tested at 10 final assay concentrations in the range from 1 × 10^−5^ M to 3 × 10^−10^ M. The final DMSO concentration in the reaction cocktails was 1% in all cases. The reaction cocktails were incubated at 30°C for 60 minutes. The reaction was stopped with 50 μl of 2% (v/v) H_3_PO_4_, plates were aspirated and washed two times with 200 μl 0.9% (w/v) NaCl. Incorporation of ^33^Pi (counting of cpm) was determined with a microplate scintillation counter (Microbeta, Wallac). All assays were performed with a BeckmanCoulter/SAGIAN™ Core System. The median value of the cpm at full activity of protein kinase in the absence of any inhibitor was defined as “high control”. The median value of the cpm in the absence of the protein kinase was defined as “low control”. As part of the data evaluation the low control value was subtracted from the high control value as well as from all “compound values”. The residual activity (in %) was calculated by using the following formula:

Res. Activity (%) = 100 X [(cpm of compound – low control) / (high control – low control)]

The residual activities for each concentration and the compound IC50 values were calculated using *Quattro Workflow V3.1.0* (Quattro Research GmbH, Munich, Germany; www.quattro-research.com). The fitting model for the IC50 determinations was “sigmoidal response (variable slope)” with parameters “top” fixed at 100% and “bottom” at 0%. The fitting method used was a least-squares fit.

### Cellular rock assay

The cellular ROCK assay was performed at ProQinase (Freiburg, Germany). The assay implements the rat smooth muscle cell line A7r5, which endogenously expresses ROCK kinases. The endogenous expression of ROCK results in a constitutive phosphorylation of the regulatory myosin light chain at Thr18/Ser19. A7r5 cells were plated in DMEM supplementary with 10% FCS in multiwell cell culture plates. Next day, medium was exchanged for serum-free medium and compounds were added for 90 min at 37°C. The final DMSO concentration was 1%. The ROCK inhibitor Y-27632 served as internal reference control. Cells treated with 100 μM Y-27362 were defined as “low control” and the corresponding mean value was set to 0%. Cells treated with DMSO alone were defined as “high control” and the corresponding mean value was set to 100%. Quantification of MLC-Thr18/Ser19 phosphorylation is assessed in 96-well plates via ELISA using a phospho-MLC-Thr18/Ser19 specific antibody and a secondary detection antibody. IC50 values were determined using GraphPad Prism 5 software with constrain of bottom to 0 and top to 100 using a nonlinear regression curve fit with variable hill slope. The equation is a four-parameter logistic equation.

### Quantification of SMAD phosphorylation

NMuMG cells plated the day before were treated for one day with TGFβ and DMSO or an inhibitor as described in the figure legends. Cells were washed with PBS and lyzed in RIPA buffer (150 mM NaCl, 2 mM MgCl, 2 mM CaCl_2_, 0.5% Na-deoxycholate, 10% glycerol, 1% NP-40, 0.1% SDS, 50 mM Tris pH8 supplementary with 1 mM DTT, 10 mM NaF, 1 mM sodium orthovanadate and protease inhibitor cocktail (Roche)). The proteins were resolved using SDS-PAGE and transferred to PVDF membrane. For quantitative analysis of SMAD phosphorylation, the membrane was probed with p-SMAD2/3 and GAPDH specific primary antibodies followed by fluorescent secondary antibodies (LI-COR Biosciences). The signal was captured and quantified with the Odyssey CLx Imaging system and ImageJ software form 3 independent experiments.

## SUPPLEMENTARY MATERIALS FIGURES AND TABLES


