# Supplementary material for: A high-content EMT screen identifies multiple receptor tyrosine kinase inhibitors with activity on TGFβ receptor
Source: Oncotarget. 2016 Mar 27;7(18):25983–6002. doi: 10.18632/oncotarget.8418 (PMC5041959; doi:10.18632/oncotarget.8418)
Supplement: Supplementary file 1 [file oncotarget-07-25983-s001.pdf]

A high-content EMT screen identifies multiple receptor tyrosine kinase inhibitors with activity on TGFβ receptor

Supplementary Materials

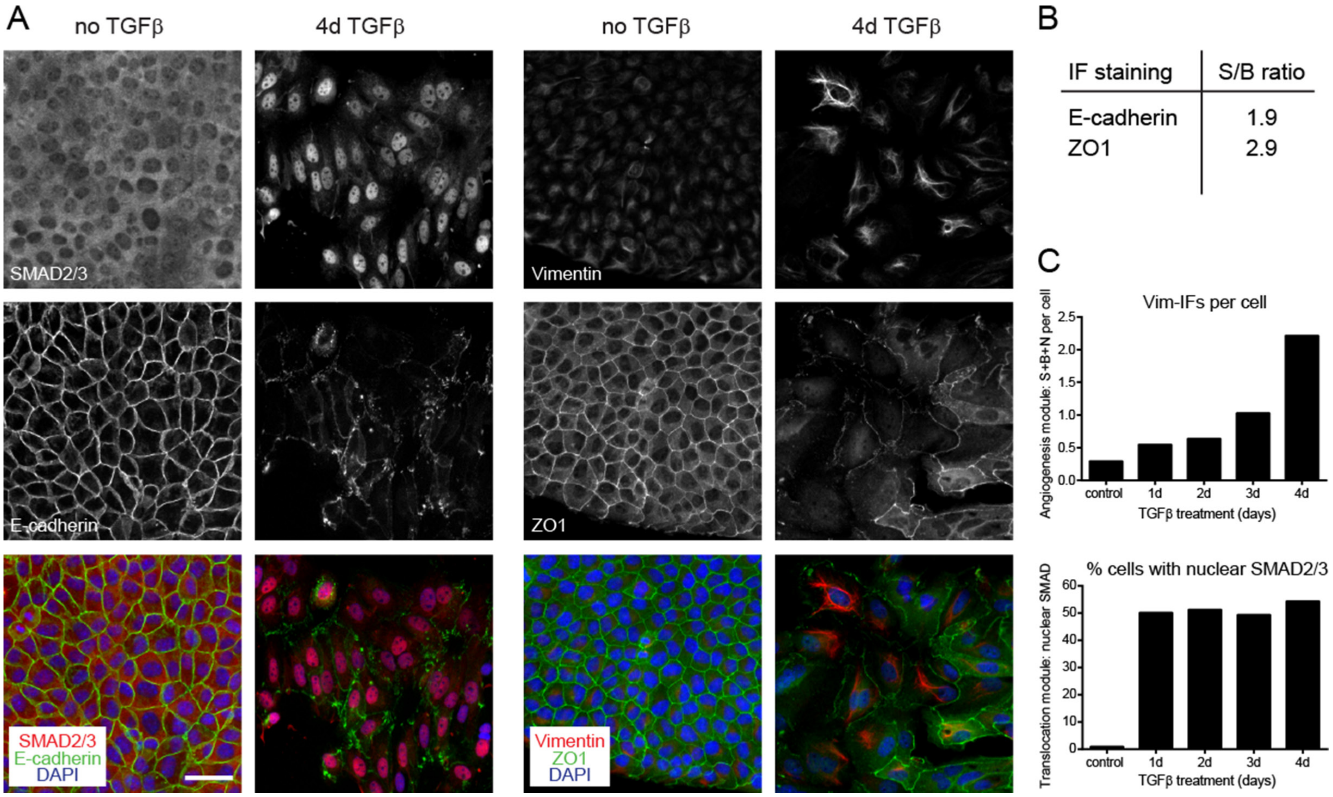

Supplementary Figure S1: Assessment of additional markers of EMT as screening readouts. (**A**) NMuMG cells were treated for four days with or without TGFβ, and the nuclear translocation of SMAD2/3 as a consequence of TGFBR activation as well as the dissolution of E-cadherin-positive adherens junctions was followed by immunofluorescence staining with specific antibodies. Vimentin was used to stain intermediate filaments and ZO1 as an epithelial marker for tight junctions. The cells were counterstained with DAPI to visualize nuclei. Scale bar, 100 µm. (**B**) Signal-to-background (S/B) ratio of E-cadherin and ZO1 immunofluorescence (IF) staining was quantified from epithelial NMuMG cells in the absence of TGFβ. Note that such low S/B ratios hamper adequate image segmentation, and no screening window between the epithelial membrane localization and the mesenchymal disintegration of these junctions was obtained in this screening setup. (**C**) NMuMG cells were treated with TGFβ for 1, 2, 3, 4 days or left untreated. The appearance of vimentin-positive intermediate filaments (Vim-IFs) per cell and the percent of cells with nuclear SMAD2/3 translocation was quantified from immunostainings with MetaXpress software. While the increase of Vim-IFs after four days of TGFβ treatment compared to untreated controls was significantly lower than the deposition of fibronectin (see Figure 1), nuclear translocation of SMAD2/3 occurred very early and as a readout would restrict the screening hits to compounds directly acting on the immediate effects of TGFBR activation by TGFβ instead of capturing the process of EMT as a whole.

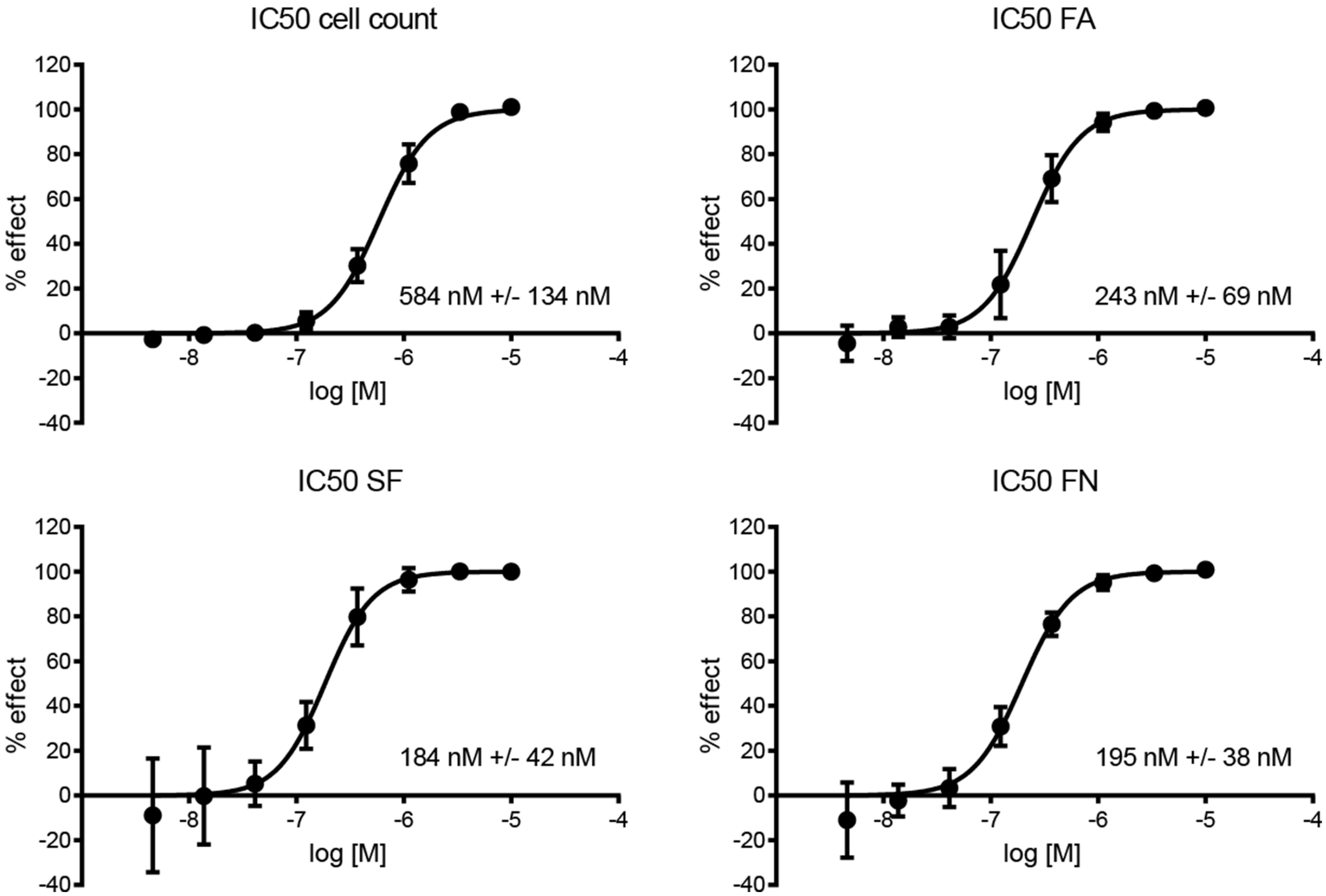

Supplementary Figure S2: Dose-dependent effect of the TGFBR inhibitor SB-431542 on the different screening readouts. Dose-response curves for the treatment of NMuMG cells with TGFβ and different concentrations of the TGFBR inhibitor SB-431542 for four days. Plotted are the log concentration values of the inhibitor (M, molar) against % effect on cell count, focal adhesion formation (FA), remodeling to stress fibers (SF) and fibronectin deposition (FN) relative to DMSO controls. IC50 values with their standard deviations are reported on the lower right of the graphs.

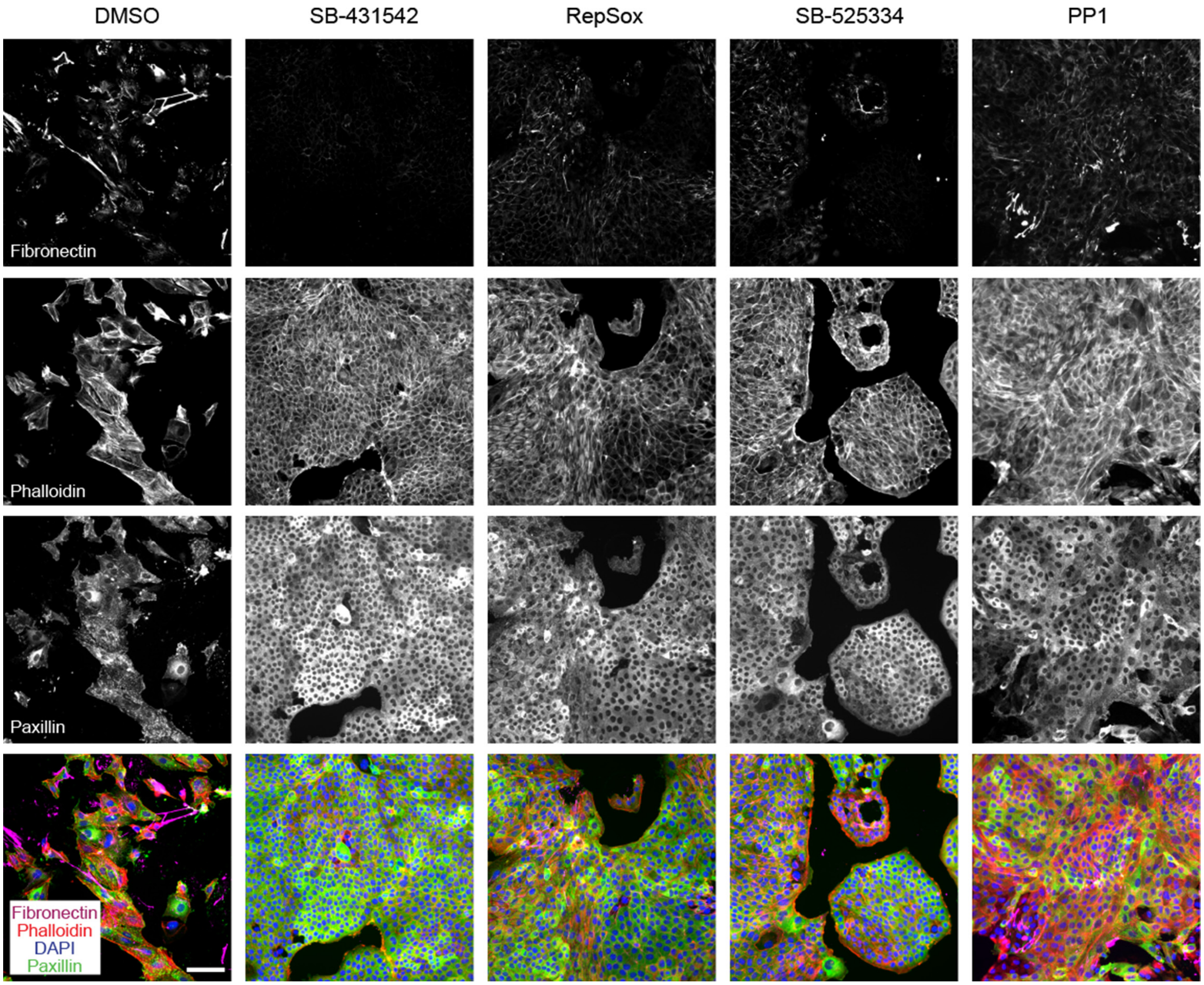

Supplementary Figure S3: NMuMG cells treated with compounds with reported activity on TGFBR stay epithelial as judged by the screening readouts. NMuMG cells were treated and stained as described in Figure 3A. SB-431542 was used at 10 µM, RepSox at 0.1 µM, SB-525334 at 1 µM and PP1 at 3 µM concentration. Scale bar, 100 µm.

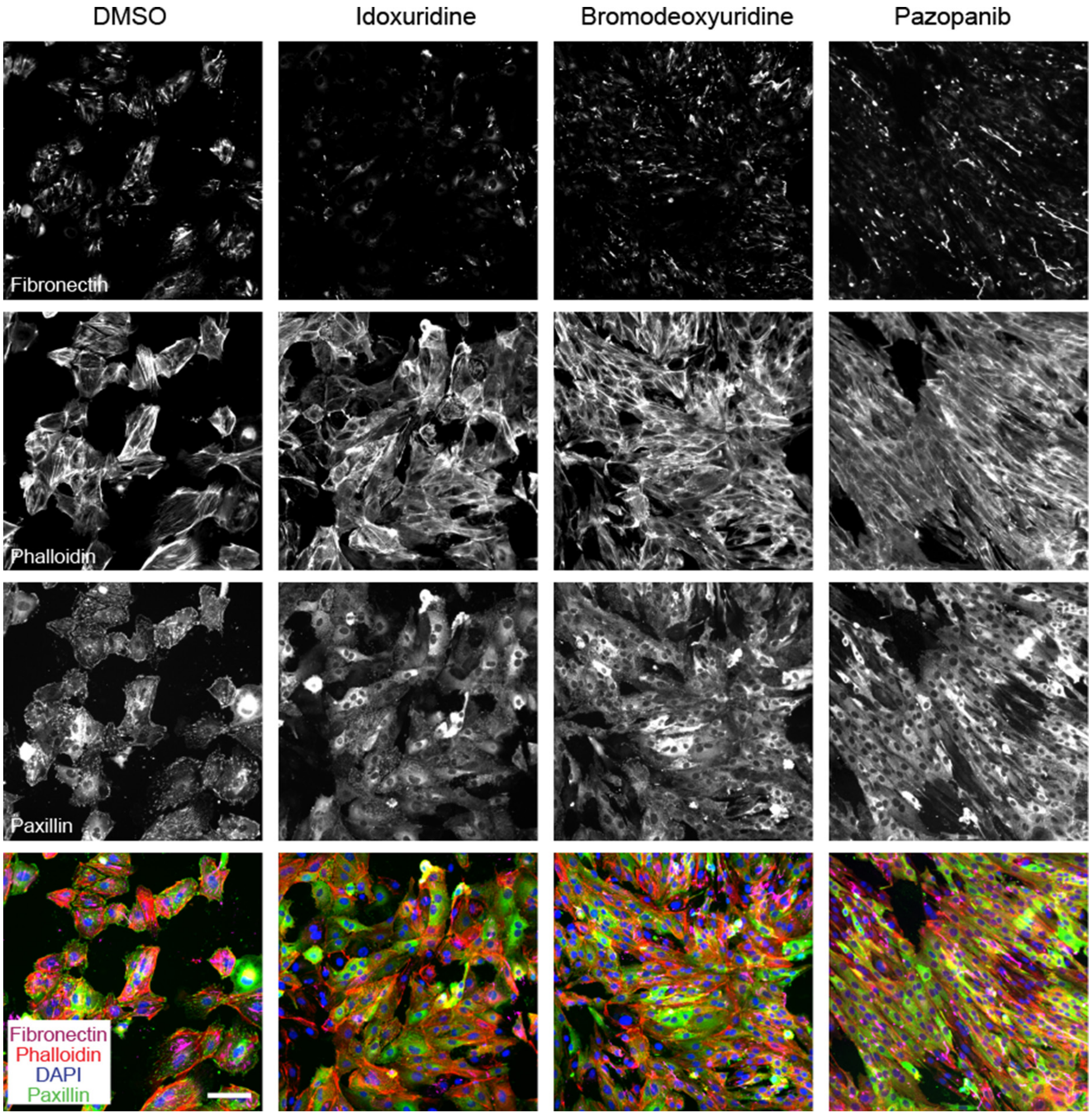

Supplementary Figure S4: Uridine analogues and pazopanib inhibit EMT progression. NMuMG cells were treated and stained as described in Figure 3A. Idoxuridine was used at 3 μM, 5-bromo-2′-deoxyuridine at 1 μM and pazopanib at 8 μM concentration. Scale bar, 100 μm.

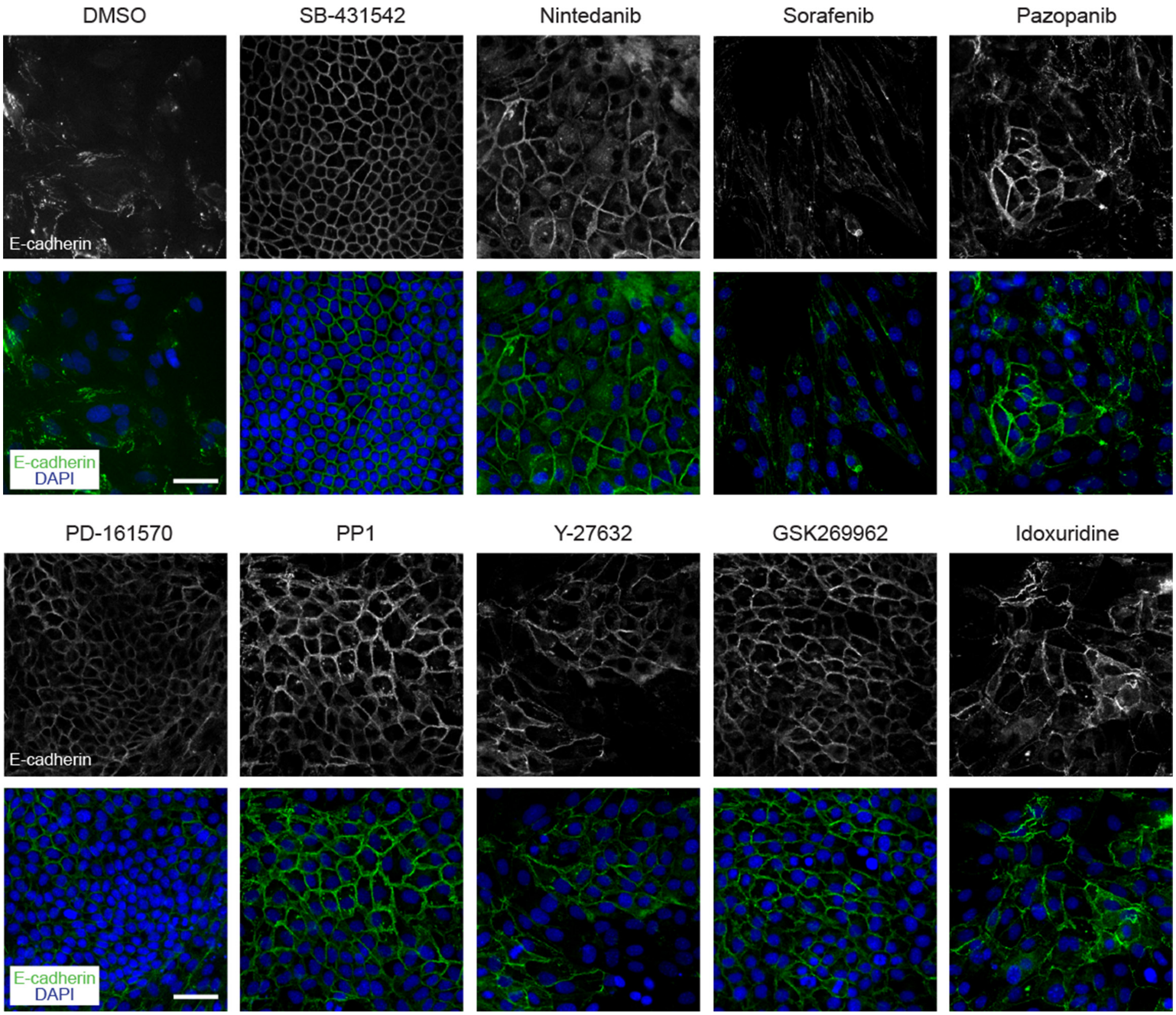

Supplementary Figure S5: E-cadherin remains at the cell membrane after TGFβ treatment in the presence of EMT inhibitors. NMuMG cells were treated for four days with TGFβ and either DMSO as a control or with a panel of inhibitors representing the different groups of EMT hits. E-cadherin staining was used as an epithelial marker to monitor EMT progression and the concomitant loss of adherens junctions. Cell nuclei were visualized with DAPI. SB-431542, nintedanib and sorafenib were used at 10 µM, PD-161570 at 1 µM, pazopanib, PP1, Y-27632 and idoxuridine at 3 µM and GSK269962 at 10 nM concentration. Scale bar, 100 µm.

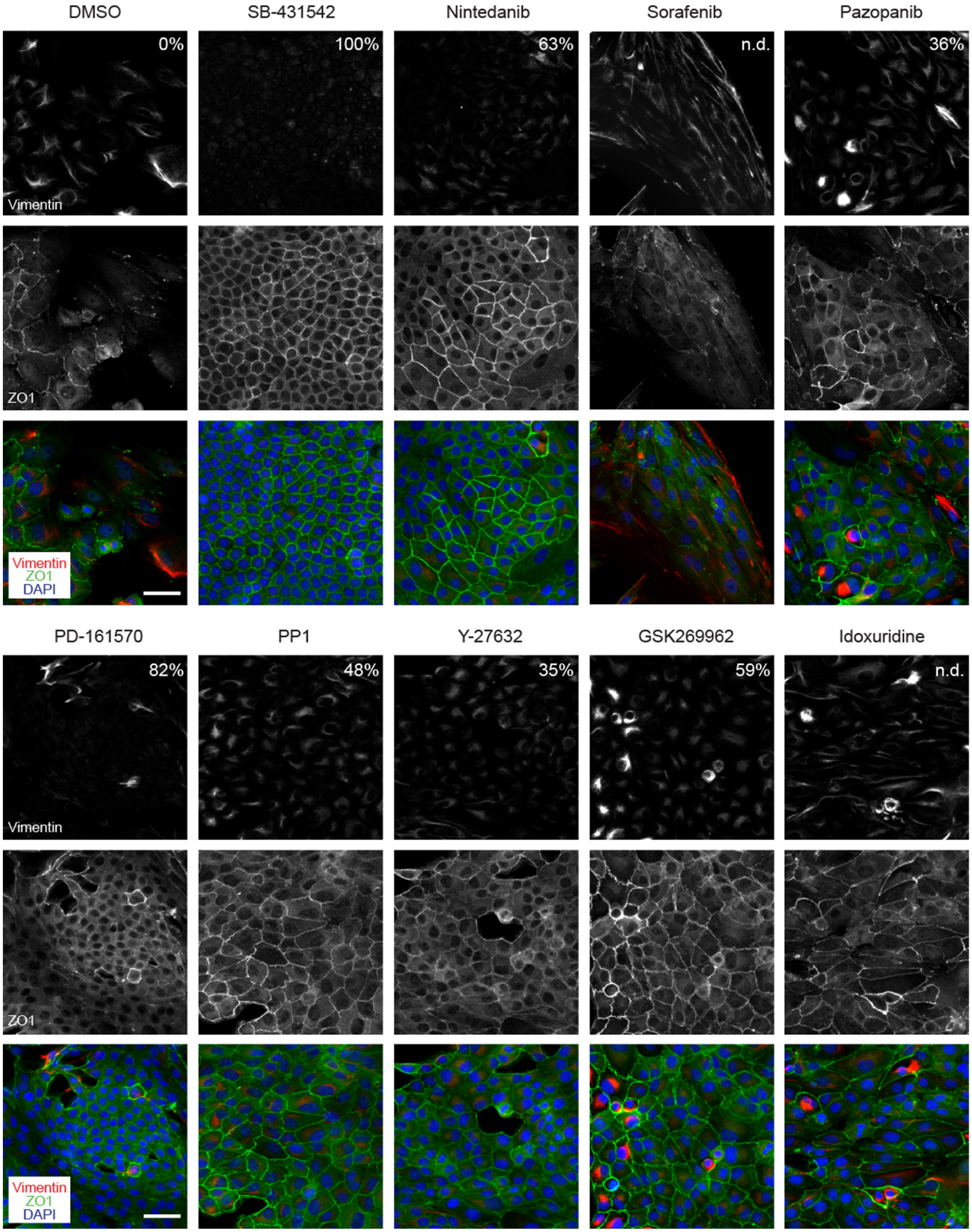

Supplementary Figure S6: The formation of vimentin intermediate filaments and the removal of ZO1 from tight junctions is blocked by the EMT inhibitors. NMuMG cells were treated for four days with TGFβ and either DMSO as a control or with a panel of inhibitors representing the different groups of EMT hits. ZO1 staining was used as an epithelial marker to monitor EMT progression and the concomitant loss of tight junctions while the formation of vimentin-positive intermediate filaments was used as a mesenchymal marker. Cell nuclei were visualized with DAPI. SB-431542, nintedanib and sorafenib were used at 10 µM, PD-161570 at 1 µM, pazopanib, PP1, Y-27632 and idoxuridine at 3 µM and GSK269962 at 10 nM concentration. The maximal effect of the inhibitors on vimentin intermediate filament formation is indicated on the top right corner of the vimentin images. The effect on the mesenchymal DMSO control was set to 0% while 100% effect corresponds to the loss of vimentin intermediate filaments after treatment with SB-431542, the TGFBR control inhibitor. No dose-response curve on vimentin intermediate filament formation was observed with idoxuridine and sorafenib, as indicated by n.d., not determined. Scale bar, 100 µm.

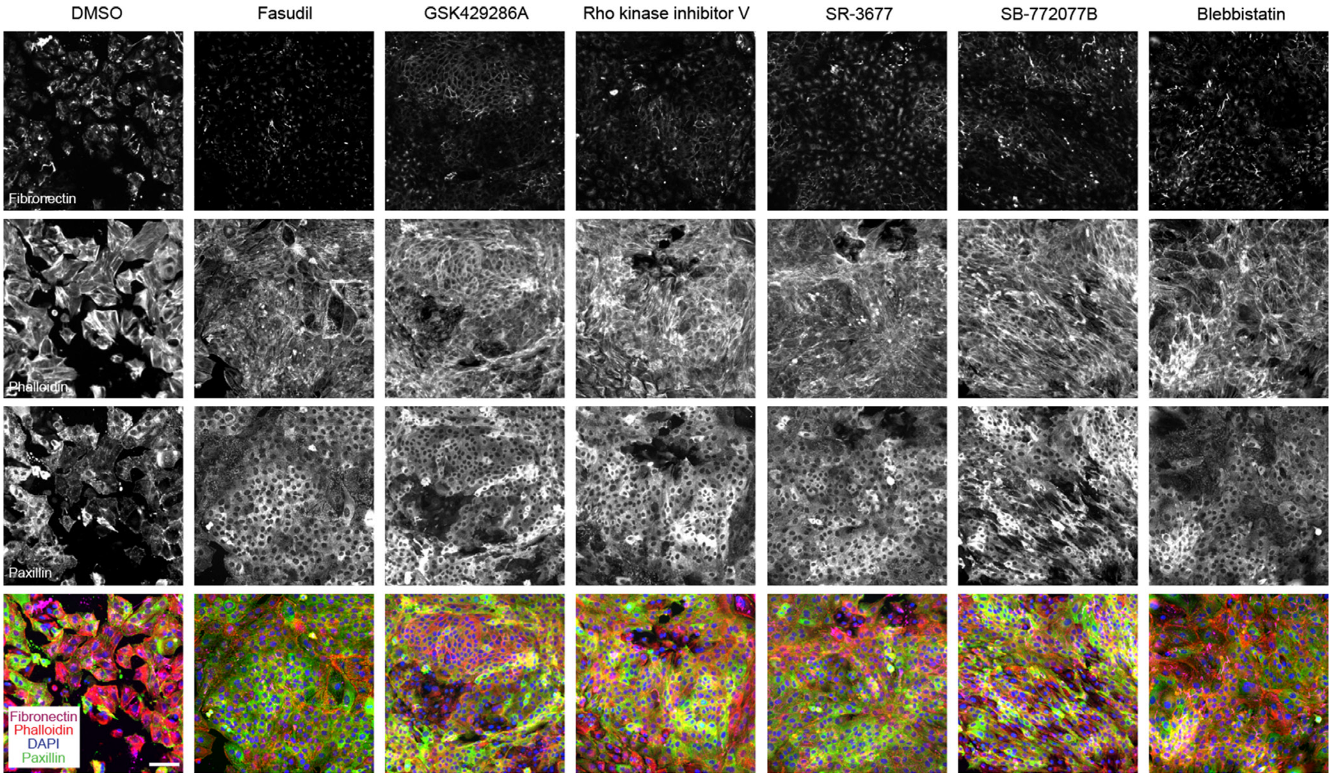

Supplementary Figure S7: ROCK pathway inhibitors block the upregulation of fibronectin, stress fibers, focal adhesion and relieve the TGFβ-induced proliferation block. NMuMG cells were treated and stained as described in Figure 3A. Fasudil was used at 10 µM, GSK429286A, Rho kinase inhibitor V and SR-3677 at 0.3 µM, SB-772077B at 0.1 µM and blebbistatin at 5 µM concentration. Scale bar, 100 µm.

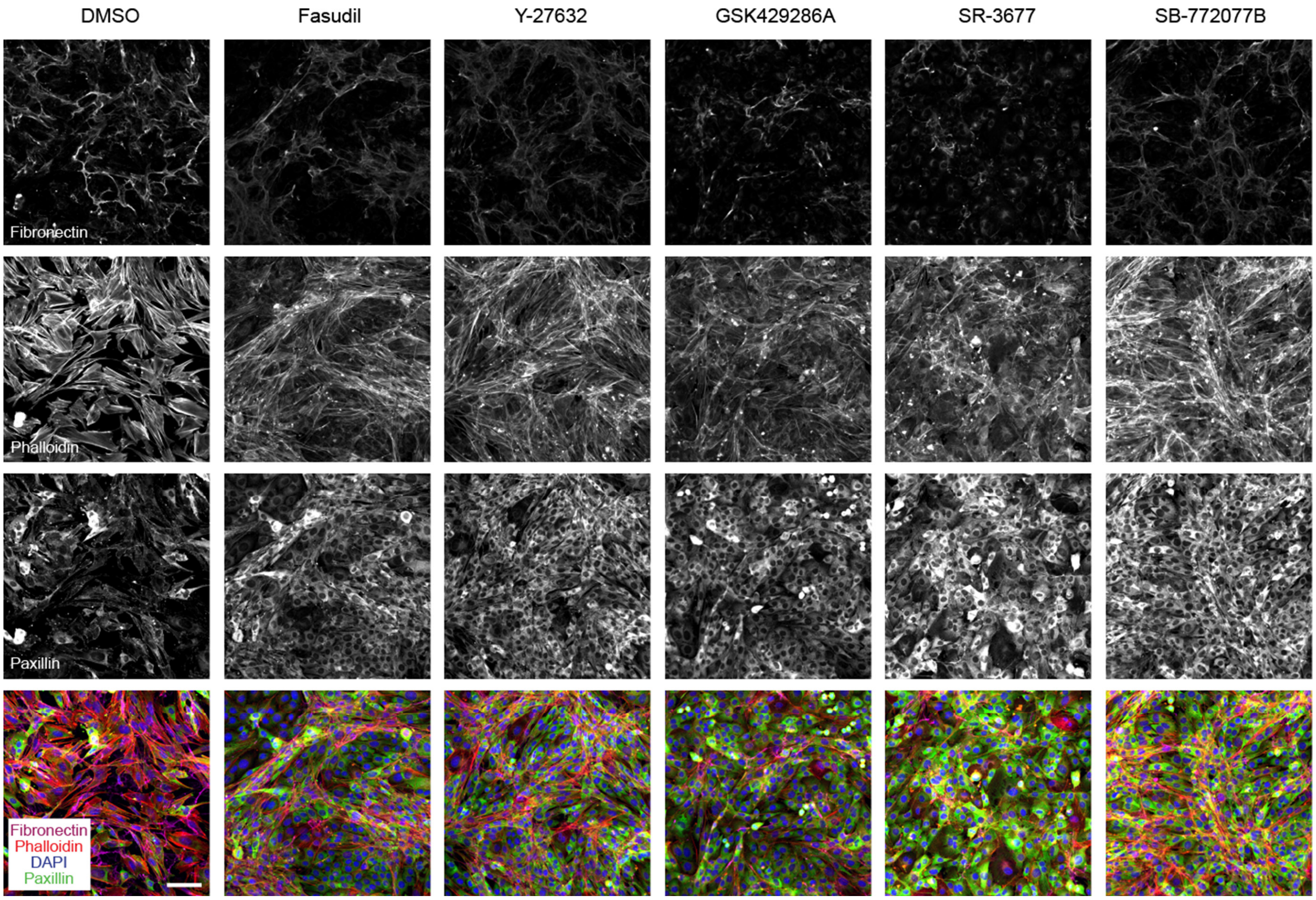

Supplementary Figure S8: ROCK inhibitors block the upregulation of fibronectin, stress fibers and focal adhesion in Py2T cells. Py2T cells were treated and stained as described in Figure 3A. Fasudil was used at 10 µM, Y-27632 at 3 µM, GSK429286A at 0.3 µM, SR-3677 at 1 µM and SB-772077B at 0.1 µM concentration. Scale bar, 100 µm.

Supplementary Table S1: Selected list of compounds with cytotoxicity

		FA/SF/FN	Tox	CCI	Emax
**Compound name**	**Reported drug targets**	**IC50 [nM]**	**IC50 [nM]**	**IC50 [nM]**	**%**
Erlotinib	EGFR	n.m.	68.5	n.m.	n.m.
Lapatinib	EGFR, HER2	n.m.	133.5	n.m.	n.m.
Gefitinib	EGFR	n.m.	105	n.m.	n.m.
AEE788	EGFR, HER2	n.m.	44	n.m.	n.m.
PP2	SRC family (LCK, FYN), TGFR1/2	n.m.	4730	n.m.	n.m.
KX2-391	SRC, pretubulin	n.m.	15	n.m.	n.m.
Dasatinib	BCR-ABL, SRC family (SRC, LCK, YES, FYN), c-KIT, EPHA2, PDGFRB	n.m.	50.9	n.m.	n.m.
PD-166326	c-ABL, c-SRC	n.m.	9810	n.m.	n.m.
5-Azacytidine	Nucleosid Analog	n.m.	1259	n.m.	n.m.
5-Aza-2′-deoxycytidine	Nucleosid Analog	n.m.	391	n.m.	n.m.
Etoposide	Topoisomerase II	n.m.	1170	n.m.	n.m.
Salinomycin		n.m.	240	n.m.	n.m.
Abamectin		n.m.	9500	n.m.	n.m.
Nigericin		n.m.	<40	n.m.	n.m.
Vatalanib	VEGFR1 (VEGFR2/3, PDGFRB, c-KIT, c-FMS)	n.m.	10585	n.m.	n.m.
Axitinib	VEGFR1/2/3, PDGFRA/B, c-KIT, (CSF-1R)	n.m.	697	n.m.	n.m.
Vandetanib	VEGFR2/3, EGFR	n.m.	208	n.m.	n.m.
Cediranib (azd2171)	VEGFR2 (VEGFR1/3, PDGFRA/B, c-KIT, CSF-1R, FGFR1)	n.m.	967	n.m.	n.m.
Sunitinib malate	PDGFRB/A, VEGFR2, c-KIT, FLT-3, CSF-1R, RET	n.m.	5100	n.m.	n.m.
Diphenyleneiodonium chloride (DPI)	NO synthase, NADPH oxidase	n.m.	453	n.m.	n.m.
Crizotinib	c-MET, ALK	n.m.	1715	n.m.	n.m.
U0126	MEK1,2	n.m.	1600	n.m.	n.m.

Compounds are selected based on their targets' or their reported role in EMT. In addition, as a comparison to the list of active compounds, cytidine analogues and receptor tyrosine inhibitors for EGFR, PDGFR and VEGFR families are shown. Listed are the compounds' name, their reported drug targets based on current literature and their effect on EMT progression as outlined in the legend of Table 1.
n.m., not measurable (no measurable effects at non-toxic concentrations).

Supplementary Table S2: ROCK pathway inhibitors have no off-target activity on TGFBR

		biochemical kinase profiling							NMuMG
		**EGFR**	**FGFR1**	**FGFR2**	**FGFR3/4**	**PDGFRA/B**	**SRC**	**TGFBR1/2**	**EMT (FA/ SF/FN)**
**Compound name**	**Reported drug targets**	**IC50 [nM]**	**IC50 [nM]**	**IC50 [nM]**	**IC50 [nM]**	**IC50 [nM]**	**IC50 [nM]**	**IC50 [nM]**	**combined IC50 [nM]**
Fasudil	ROCK1 & 2	ia	ia	ia	ia	ia	ia	**ia**	3023.3
Y-27632 2HCl	ROCK1 & 2	ia	ia	ia	ia	ia	ia	**ia**	1717.3
GSK429286A	ROCK1 & 2	ia	ia	ia	ia	ia	ia	**ia**	157.7
Rho kinase inhibitor V	ROCK1 & 2	ia	2945	7107	ia	ia	ia	**ia**	456.7
SR-3677	ROCK1 & 2	ia	ia	ia	ia	ia	ia	**ia**	123.3
GSK269962	ROCK1 & 2	ia	ia	ia	ia	ia	ia	**ia**	3.3
SB-772077B	ROCK1 & 2	ia	ia	ia	ia	ia	ia	**ia**	44.7
CAY10622	ROCK1 & 2	ia	ia	ia	ia	ia	ia	**ia**	ia
Blebbistatin	Myosin II	ia	ia	ia	ia	ia	4370	**ia**	3363.3

ROCK inhibitors and the myosin II inhibitor blebbistatin were profiled for potential off-target activity towards kinases of the EGFR, FGFR, PDGFR, SRC and TGFBR families, and the IC50 of *in vitro* inhibitory activity was compared to their IC50 for the EMT readouts. Combined IC50 for EMT parameters represents the mean of IC50 values obtained for FA, SF, FN patterns. Note that no activity towards TGFBR1 and TGFBR2 was measured.
FA, focal adhesions; SF, stress fibers; FN, fibronectin patches; ia, inactive (> 10000 nM).
